# Development of *Ac*- and *Ds*-tagged starter lines for large-scale transposon-mutagenesis in tomato

**DOI:** 10.1371/journal.pone.0335612

**Published:** 2025-11-19

**Authors:** Alka Kumari, Rachana Ponukumatla, Arun Kumar Pandey, Yellamaraju Sreelakshmi, Rameshwar Sharma

**Affiliations:** Repository of Tomato Genomics Resources, Department of Plant Sciences, University of Hyderabad, Hyderabad, India; Texas Tech University, UNITED STATES OF AMERICA

## Abstract

Tomato (*Solanum lycopersicum*), a model for fleshy fruit ripening, is predicted to possess ~40,000 genes based on *in silico* homology-based annotation. However, the functional roles of most annotated genes remain unvalidated. Transposon-tagged mutagenesis offers a powerful strategy for functional genomics, enabling gene identification through phenotypic analysis and activation tagging. Yet, the lack of an efficient *in planta* transformation system has limited large-scale transposon mutagenesis in tomato. To overcome this limitation, we developed two tomato starter lines, each harboring a maize transposon element: the Dissociation (*Ds*) element and its corresponding Activator (*Ac*) transposase. Crossing these lines induced *Ac*-mediated transposition of *Ds* in the F_1_ generation. In the F_2_ progeny, we tracked the excision and reintegration of *Ds* across the genome. The *Ds* insertions were distributed across multiple chromosomes, confirming unlinked transposition. Sequencing of flanking regions revealed random integration into genic, intergenic, and promoter regions. Our study establishes a platform for transposon-tagged mutagenesis in tomato, providing a valuable resource for large-scale functional gene validation.

## Introduction

The impending challenges of climate change and a rapidly growing global population have underscored the need to double food production by 2050 [**1]**. While cereals remain the primary source of caloric intake, there is a growing impetus to enhance both yield and nutraceutical content in vegetable crops. Tomato, a widely cultivated crop worldwide, is rich in several nutraceutical compounds that are largely absent in cereals [**2**]. However, as with many domesticated crops, selective breeding in tomato has led to a decline in nutritional quality, flavor, and disease resistance due to genetic erosion [3,4]. Genome resequencing of numerous tomato cultivars and their wild relatives has revealed the significant loss of genetic diversity in modern tomato varieties resulting from domestication [5,6].

The genetic base of domesticated crops can be broadened either through the introgression of chromosomal segments from wild relatives or by inducing de novo genetic diversity via mutagenesis [**7**]. Before the genomics era, mutagenesis-induced diversity was exploited by visually selecting mutants with desirable traits, followed by backcrossing to parental lines. The advent of crop genome sequencing has greatly enhanced this process by enabling the use of molecular markers to guide introgression [**8**]. It has also facilitated the functional genomic analysis of genes disrupted by chemical or radiation mutagens, T-DNA insertions, transposon tagging, and the latest CRISPR-Cas9 genome editing. Each of the above mutagenesis approaches has unique strengths and limitations for functional genomics. CRISPR-Cas9, for example, enables highly efficient and targeted editing of specific loci. However, its utility can be compromised by off-target mutations at similar sequences and reduced efficiency in polyploid species due to allelic sequence mismatches [9]. In contrast, traditional mutagenesis techniques, which generate random genome-wide mutations, are excellent for generating wide phenotypic variability (6). Among the traditional mutagenesis approaches, transposons hold a prominent position due to their ability to generate large collections of gene knockouts. The utility of the autonomous Activator (*Ac*) transposable element from maize, first discovered by **McClintock** [**10**], and its nonautonomous derivative, Dissociation (*Ds*), for gene tagging and insertional mutagenesis is well established [**11**]. Notably, *Ac/Ds* elements function effectively in heterologous systems, as the *Ac*-encoded transposase is enzymatically active and capable of mobilizing both autonomous and nonautonomous elements.

Compared to T-DNA mutagenesis, the *Ac/Ds* transposon system offers several advantages. First, while T-DNA mutagenesis typically requires large-scale transformations, the *Ac/Ds* system can generate numerous transposon-tagged lines from just a few primary transformants. Second, *Ds* insertions can occur either in somatic tissues or during gametogenesis. The former is valuable for activation tagging, whereas the latter leads to novel germline insertions. Third, because *Ds* is a nonautonomous element, stable *Ds* mutants can be obtained in subsequent generations as the *Ac* element segregates away.

The maize *Ac/Ds* system has been successfully utilized for gene tagging and insertional mutagenesis in several plant species, including *Arabidopsis* [12,13], rice [14,15], and tomato [16,17]. In tomato, the *Ac/Ds* elements enabled the identification of *CF-9*, a gene involved in resistance to the fungal pathogen *Cladosporium fulvum* [**18**]. The *DCL* gene, required for chloroplast development and palisade cell morphogenesis, and the *FEEBLY* gene, involved in general plant development, were also identified through *Ac/Ds*-mediated gene tagging [19,20].

Two main strategies have been employed to generate *Ac/Ds*-based insertional mutant lines. The first involves placing both the *Ac* and *Ds* elements within the same construct [**14**], enabling transposition without the need for crossing. However, isolating plants that retain only the *Ds* element requires multiple rounds of screening to distinguish lines in which only *Ds* remains at the new site, but *Ac* has segregated away. This approach was previously applied by **Meissner et al.** [**16**] and **Carter et al**. [**17**] to develop *Ds*-tagged tomato lines. The second strategy places *Ac* and *Ds* on separate constructs, which are independently introduced into starter lines via transformation [**15**]. Crossing these lines activates *Ac*-mediated transposition of *Ds* to new genomic locations, allowing for straightforward selection of *Ds*-only progeny.

Using separate starter lines for *Ac* and *Ds* offers several advantages over the single-construct approach. It allows controlled activation of *Ds* transposition through crossing, eliminates the need for tissue culture in generating large populations of tagged lines, and minimizes *Ac*-induced instability at *Ds* insertion sites, an issue when both elements are present in the same construct.

Taking cognizance of the advantages described above, we generated an insertional mutant population in tomato using the second strategy, in which two independent sets of starter lines were developed, one carrying the *Ac-*transposase and the other containing the *Ds*-transposon. The *Ac*-transposase line was marked with GFP as a reporter, while the *Ds* element was tagged with both GFP and RFP. Upon mobilization to a new genomic site, the *Ds* element excised itself, carrying RFP but leaving GFP at the original insertion site. Selection of *Ds*-transposed plants was simplified by screening for individuals that were GFP-negative and RFP-positive, enabling efficient identification of *Ds*-only plants. This two-starter line system offers greater flexibility and reliability compared to single-construct systems for generating large insertional mutant populations.

## Materials and methods

### Plant material, plasmids, and growth conditions

Seeds of tomato (*Solanum lycopersicum* cv. Arka Vikas) were obtained from the Indian Institute of Horticulture, Bangalore, India. Seeds were surface-sterilized with 4% (v/v) sodium hypochlorite for 10 minutes and subsequently sown in coconut peat (Sri Balaji Agro Services, India). Seedlings were grown in a growth room at 25 ± 1°C (40–50% RH) under a 16 h/8 h light/dark cycle. They were later transferred to a greenhouse and cultivated under natural day/night conditions (50–70% RH). For visualization of fluorescence in transgenic seedlings, seeds were germinated and grown in a dark room maintained at 25 ± 1°C. Plasmids pSSZ40, pSSZ36, and pSQ3 [15,21] were generously provided by Prof. Venkatesan Sundaresan, Department of Plant Biology, University of California, USA. All experimental procedures adhered to relevant institutional, national, and international guidelines and regulations.

### Vector construction

#### Preparation of *Ac* vector.

The *Ac* vector was constructed using pBINPLUS as the backbone, modified from the plasmids pSSZ36 and pSSZ40 (S1, S2 Fig., See S1 File **for all raw images for gel/blots**). First, the *CaMV35S::Ac* cassette was excised from pSSZ36 as a 4.7 Kb BamHI/SalI fragment, containing a minimal *CaMV35S* promoter driving *Ac* gene expression in plants. This fragment was then cloned into the binary vector pBINPLUS (S2A, B Fig.). In parallel, the *ZmUbi1::sGFP* expression cassette was excised from pSSZ40 as a 3 Kb BamHI/KpnI fragment and inserted into the pBINPLUS backbone (S2C-G Fig.). To enhance reporter gene expression in tomato, a dicot species, the *ZmUbi1* promoter was replaced with the *CaMV35S* promoter. The *CaMV35S* promoter was excised from pBI121 using HindIII/XbaI, and the resulting 2 Kb HindIII/KpnI fragment, comprising the reporter gene cassette (*CaMV35S::sGFP::NosT*), was cloned into the pBINPLUS vector alongside the *Ac* cassette (S2H–K Fig.).

#### Preparation of *Ds* vector.

The original *Ds* construct was designed for optimal expression in monocot plants. To adapt it for use in tomato, the *Ds* vector was reconstructed using pBINPLUS as the backbone and the *Ds* element from the plasmid pSQ3. A 6.7 Kb SacI insert containing *Ds* 5′ and 3′ sequences flanking a 4X *CaMV35S* enhancer and the *ZmUbi1::RFP/DsRed* cassette was excised from pSQ3 (S3, S4A-C Fig.) and subsequently cloned into the pBINPLUS vector, which already harbored the GFP cassette (S4D-F Fig.). To enhance RFP expression in tomato, the *ZmUbi1* promoter driving RFP expression was replaced with the *AtUbi3* promoter.

### Genetic transformation

Tomato genetic transformation was performed using the *Agrobacterium*-mediated co-cultivation method, as described by **Sharma et al. [**22**],** with minor modifications. Briefly, explants were harvested from 7-day-old seedlings and incubated in an *Agrobacterium* suspension. After co-cultivation, the explants were further incubated for 48 hours before being transferred to selection medium. Notably, the *Agrobacterium* strain C58C1 was used in this study instead of the AGL1 strain specified in the original protocol. The composition of the MS medium and the hormone concentrations applied at various regeneration stages are detailed in S1 Table. Regenerated plants were acclimatized and potted, then grown in a greenhouse until maturity. A stepwise summary of explant survival and transformation frequency, resulting in 24 independent *Ac-TPase* and 20 *Ds* transgenic lines, is provided in S2 Table.

### Genomic DNA isolation and PCR analysis

Genomic DNA was extracted from tomato leaves following the protocol described by **Sreelakshmi et al. [**23**].** For PCR amplification, 50–100 ng of genomic DNA was used per reaction. PCR was performed using a Bio-Rad thermocycler under cycling conditions optimized for each primer set. To confirm the presence of transgenes, primers specific to the *Ac* and *NPTII* genes were used (S5A–C Fig.). Transgene segregation in the T_1_, T_2_, and T_3_ generations was assessed using a PCR-based approach. This involved a forward primer targeting the *NPTII* gene and a reverse primer specific to the *NOS* terminator, yielding a ~ 750 bp product indicative of the transgene (S5 Fig.). In the F_2_ generation, segregation of *Ac/Ds* elements was further monitored using GFP- and RFP-specific primers. The complete list of primers used in this study is provided in S3 Table.

### Southern blot analysis

Genomic DNA was extracted from young leaf tissues of putative transgenic tomato plants using the protocol described by **Sreelakshmi et al. [**23]. Approximately 10–12 µg of genomic DNA was digested with the appropriate restriction enzymes, and the digested DNA was resolved on a 0.8% (w/v) agarose gel in 1 × TAE buffer at 50 V for 16–18 hours to allow adequate separation of high-molecular-weight fragments. Following electrophoresis, the gel was sequentially treated (depurination, denaturation, and neutralization solutions) to prepare the DNA for nylon membrane transfer through capillary action: Southern blotting was performed following the standard protocol described by **Sambrook et al. [**24**].** For the detection of the *Ac* transgene in transgenic lines, a 4.7 kb BamHI/SalI fragment containing the *Ac* element from the pSSZ36 plasmid was used as a probe. To assess the presence and copy number of the *Ds* transgene, a 780 bp fragment of the *NPTII* gene derived from the pBINPLUS vector was used as a probe. Probes were labeled and hybridized according to standard protocols (S6 Fig.).

### GFP/RFP fluorescence assay

The *Ac* and *Ds* constructs used to generate the insertional mutant population contain two fluorescent reporter genes: *sGFP/GFP* and *DsRed/RFP*. Putative transgenic plants, untransformed controls (as a negative control), and homozygous *GFP* plants in the Moneymaker background [**25**] (as a positive control) were examined for fluorescent protein expression. In the T_0_ generation, leaves of transgenic plants were examined under a confocal microscope, and images were captured using a Leica TCS SP2 Confocal Laser Scanning Microscope. sGFP fluorescence was visualized with excitation/emission at 450/535 nm.

In the subsequent T_1_/T_2_/T_3_ generations, 5-day-old dark-grown seedlings from *Ac* and *Ds*-transformed plants were screened for sGFP and DsRed fluorescence to detect mobilized Ds transposable elements. This screening was conducted on a Kodak imaging station using excitation/emission wavelengths of 450/535 nm for sGFP and 572/610 nm for DsRed. Fluorescence imaging and analysis were performed with Bruker MI Project software for high-resolution visualization. The resulting images were processed and displayed as either grayscale or pseudo-colored heatmaps to improve signal contrast and facilitate the identification of fluorescent expression patterns.

### Kanamycin painting

A kanamycin leaf painting assay was performed to assess transgene segregation across successive generations and to monitor *in vivo* transgene expression. Leaflets from the 7^th^ or 8^th^ node of one-month-old transgenic and wild-type plants were selected for the assay. Three leaves per plant were tagged, and the adaxial (upper) surface was painted with 250 µg/mL kanamycin solution. After ten days of incubation, the leaves were examined for the appearance of a bleached phenotype, indicative of kanamycin sensitivity.

### Determination of the T-DNA insertion sites

To isolate the flanking genomic sequences of *Ds* insertions, we used a modified Thermal Asymmetric Interlaced PCR (TAIL-PCR) method called Fusion Primer and Nested Integrated PCR (FPNI-PCR) [26]. This nested, three-step PCR approach was optimized to enhance specificity and product yield. The primary (first-round) PCR was performed in a 20 μL reaction volume containing 2 μL of genomic DNA, 0.2 μM of the gene-specific primer Ds1, and 1.0 μM of a fusion arbitrary degenerate primer. The reaction was run for 18 cycles using the annealing temperatures specified by **Sharada et al.** [27]. Subsequent secondary and tertiary (second and third-round) PCRs replaced the initial primers with the nested gene-specific primers Ds2 and Ds3, paired with the adaptor-specific primers FSP1 and FSP2, respectively. We optimized annealing temperatures for the degenerate primers and the concentration of genomic DNA according to **Sharada et al.** [27] (S4 Table), which successfully amplified the flanking regions of transposed Ds elements. The final PCR amplicons were resolved on agarose gels, and products of the expected size were excised and purified. These products were then either sequenced directly or cloned into the pJET vector for Sanger sequencing. The resulting sequences were filtered, aligned, and analyzed using methods adapted from **Sharada et al.** [27].

Furthermore, to capture *Ds* transposition sites not isolated by FPNI-PCR, we performed inverse PCR (iPCR) as described by **Han et al.** [28] (S5 Table). This technique utilizes circularized DNA templates and outward-facing primers, in contrast to conventional PCR, where primers face inward. Briefly, 25–50 ng of genomic DNA was digested with an appropriate restriction enzyme for 3 hours. The digested DNA was either ethanol-precipitated and resuspended in water or used directly for ligation following heat inactivation. The fragments were self-ligated for 30 minutes to form circular templates. Approximately 20–50 ng of the ligated DNA was then used as a template for PCR with outward-facing primers. The resulting PCR products were separated by agarose gel electrophoresis, and bands of the expected size were excised, gel-purified, and cloned into the pJET 2.0 vector using the Blunt End Cloning Kit (Thermo Fisher Scientific) for subsequent sequencing and analysis.

## Results

### Strategy for transposon mutagenesis using *Ac/Ds* system

For transposon mutagenesis in tomato, we employed a strategy involving two independent parental lines, one carrying the *Ac* transposase (*Ac*-Activator) and the other harboring the *Ds* transposon (*Ds*-Dissociation). It was hypothesized that crossing these two lines would result in excision and reinsertion of the *Ds* element in the F_1_ progeny. The F_2_ generation could then be analyzed to identify plants in which the *Ds* element had mobilized to a new genomic location in the absence of the *Ac* transposase. To enable efficient screening at the seedling stage, we used the reporter genes GFP (green fluorescent protein) and DsRed/RFP (red fluorescent protein), which allow for detection via fluorescence imaging or PCR. S7 Fig. illustrates the sequential steps involved in *Ac/Ds* mutagenesis of tomato using this strategy.

### Modifications of *Ac* and *Ds* constructs and raising of *Ac/Ds* parental lines

**Fig 1:**
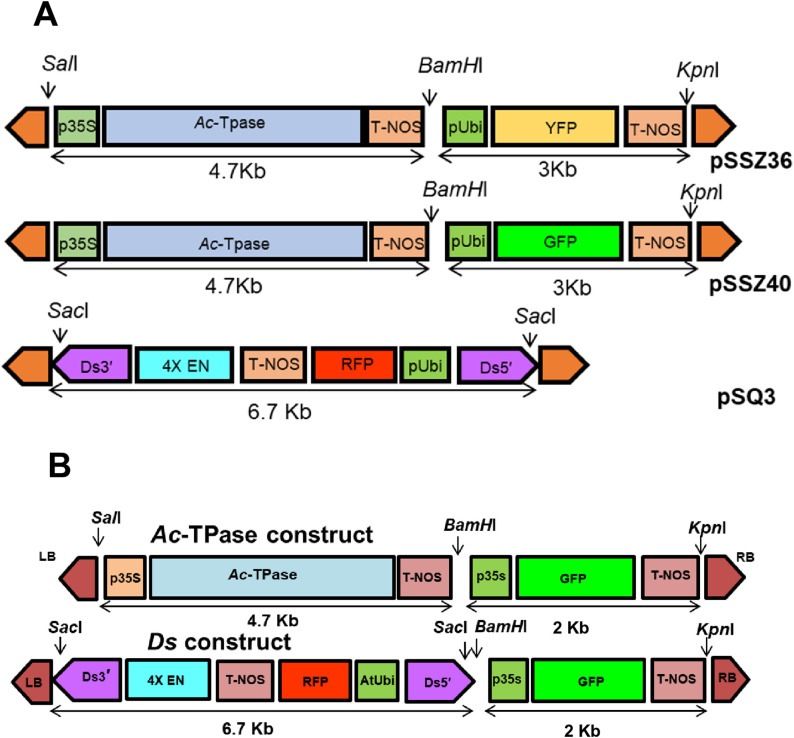
Constructs used for generating transposon-tagged lines in tomato. A. *Ac* and *Ds* constructs used for insertional mutagenesis in rice. B. Modified *Ac* and *Ds* constructs adapted for insertional mutagenesis in tomato. **Abbreviations:** *p35S*: CaMV 35S promoter; *Ac*-*TPase*: Ac transposase; T-NOS: Nopaline synthase terminator; pUbi: Maize ubiquitin 1 promoter; GFP: Green fluorescent protein gene; YFP: Yellow fluorescent protein gene; 4X EN: 4X 3CaMV5S enhancers; *Ds3*′: 3′ end of the *Ds* element; *Ds5*′: 5′ end of the *Ds* element; At-Ubi: *Arabidopsis thaliana* ubiquitin 3 promoter.

The constructs pSSZ36, pSSZ40, and pSQ3 (derived from pSQ2), which served as base vectors in this study, were originally designed for rice and were therefore not suitable for tomato (**Fig 1A**). These vectors contained monocot-specific promoters and antibiotic resistance genes optimized for rice [15,16,21]. To adapt them for tomato, the monocot-specific regulatory elements were replaced with dicot-specific promoters and selectable markers to ensure efficient gene expression and transformation in tomato (**Fig 1B**).

The *Ac* construct included an immobilized *Ac*-TPase gene, while the *Ds* construct, derived from the wild-type *Ac*-TPase element, contained a nonautonomous *Ds* element with 1785 bp at the 5′ end and 222 bp at the 3′ end (**Fig. 1**). Additionally, the *Ds* construct carried a 4X enhancer (4X EN) sequence for activation tagging. S1 and S3 Figs. illustrate the stepwise replacement of the maize ubiquitin promoter with the *Arabidopsis* ubiquitin promoter, and the substitution of the hygromycin resistance gene with a kanamycin resistance gene. S2 and S4 Figs. show the stepwise modification of the *Ac* construct for the insertion of a GFP reporter gene, and of the *Ds* construct for the insertion of both GFP and RFP reporter genes.

The modified *Ac* and *Ds* vectors were introduced into tomato cv. Arka Vikas via *Agrobacterium*-mediated genetic transformation. In the T_0_ generation, 24 *Ac* lines (Ac-1 to *Ac-24*) and 20 *Ds* (*Ds-1* to *Ds-20*) lines were selected. In the T_1_ generation, an alphabetical suffix was added to the *Ac* lines and a numerical suffix to the *Ds* lines to denote the specific parental line and plant number. Progeny from these T_1_ plants were advanced through successive generations for further analysis. For crossing the starter lines, T_4_ progeny of the *Ac* lines and T_2_ progeny of the *Ds* lines were used.

### Reporter gene expression in *Ac* and *Ds* lines

**Fig 2:**
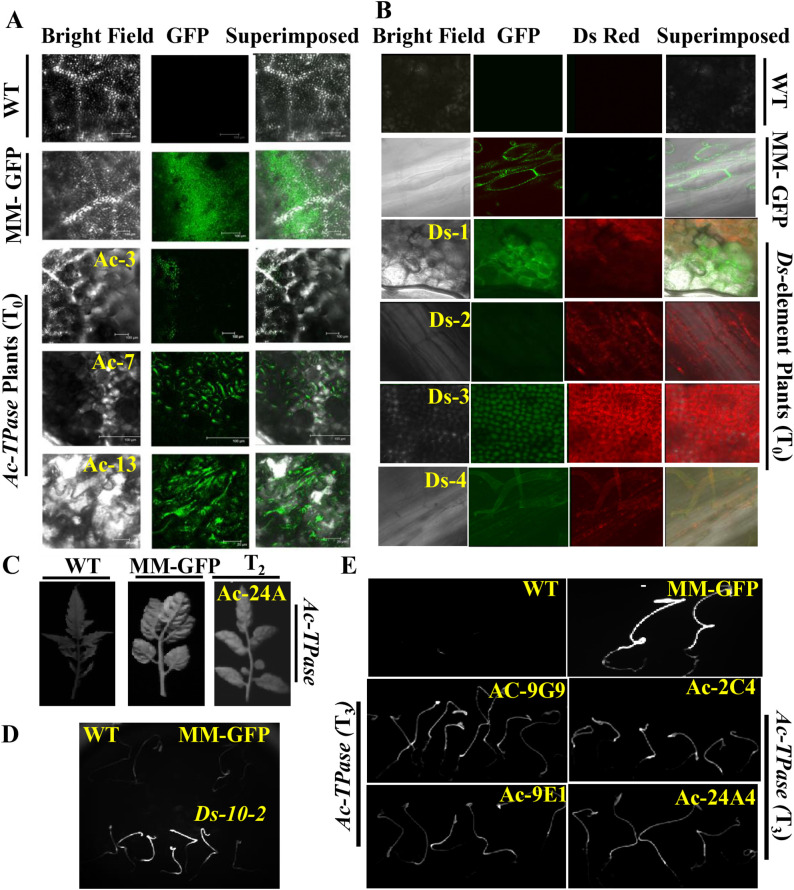
Imaging of GFP and RFP fluorescence in *Ac-* and *Ds-* plants. Wild-type (WT) plants (cv. Arka Vikas) and transgenic GFP-expressing plants in the Moneymaker background were used as negative and positive controls, respectively. The emission of GFP and RFP fluorescence confirms the presence and expression of the respective transgenes. **A-B.** Confocal laser scanning microscopy images showing GFP (**A**) and DsRed/RFP (**B**) expression in T_0_ *Ac* and *Ds* lines. C. Top panel: images of leaves taken with a GFP filter; bottom panel: corresponding bright-field images. The blue fluorescence indicates GFP signal saturation. *Ac-24A* T_2_ is a Southern-positive line. D. Top panel: images of WT (left) and MM-GFP (right) seedlings under an RFP filter. Bottom panel: RFP fluorescence in Southern-positive *Ds-10-2* T_1_ seedlings. E. Top panel: images of WT (left) and MM-GFP (right) seedlings under a GFP filter. Bottom panel: GFP fluorescence in seedlings of *Ac* T_3_ lines. Images in panels **C–E** were captured using the Kodak imaging station.

We screened the *Ac* and *Ds* lines using GFP/RFP fluorescence visualization (**Fig 2**). Additionally, a kanamycin resistance assay and PCR amplification were employed to identify transgenic lines. Plants were categorized as resistant or sensitive to kanamycin based on the absence or presence of leaf bleaching, respectively (S8 Fig., S6 Table). The transgenicity of kanamycin-resistant lines was further confirmed through PCR amplification of the transgenes (S5 Fig.). Integration of the transgene was verified by Southern blot analysis (S6 Fig).

In the T_0_ generation, *Ac* plants exhibited distinct green fluorescence, while *Ds* plants showed both green and red fluorescence. Untransformed control plants displayed no fluorescence (**Fig 2A**-B). In *Ds* T_0_ plants, GFP and RFP fluorescence was observed in the leaves (**Fig 2B**). GFP expression in *Ac* lines was further confirmed in the T_2_ generation (leaves, **Fig 2C**) and T_3_ generation (seedlings, **Fig 2E**). Dark-grown T_2_ *Ds* seedlings exhibited RFP fluorescence (**Fig 2D**). However, the intensity of GFP and RFP fluorescence varied among different *Ac* and *Ds* lines, with some lines exhibited a visibly weaker fluorescence signal.

To reduce the risk of missing *Ds*-mobilized lines due to weak fluorescence, the F_2_ population was also screened using PCR amplification of the *GFP* and *RFP* genes. Based on the combined results of Southern blotting, fluorescence imaging, and PCR assays, seven *Ds* lines and three *Ac* lines were selected for further analysis.

### Generation of *Ds*-tagged population

To generate the F_1_ population, different combinations of *Ac* (female ♀) and *Ds* (male ♂) starter lines, each harboring a single transgene copy, were manually crossed. Two independent sets of crosses were performed. The first set, including *Ds-2 *×* *Ac-9E1, Ds-9* *×* *Ac-9E1, Ds-6* *×* *Ac-9E1, Ds-8* *×* *Ac-24A3, Ds-3* *×* *Ac-2C4, and Ds-1* *×* Ac-9G9,* was analyzed for flanking sequence using FPNI-PCR (*F*usion *P*rimer and *N*ested *I*ntegrated PCR). The second set, consisting of *Ds-10–2 *×* *Ac-24A, Ds-10–8* *×* *Ac-9G, and Ds-10–1* *×* Ac-9G,* was analyzed using inverse PCR. The launch sites for *Ds-2, Ds-4,* and *Ds-10–2* are listed in S7 Table.

**Fig 3:**
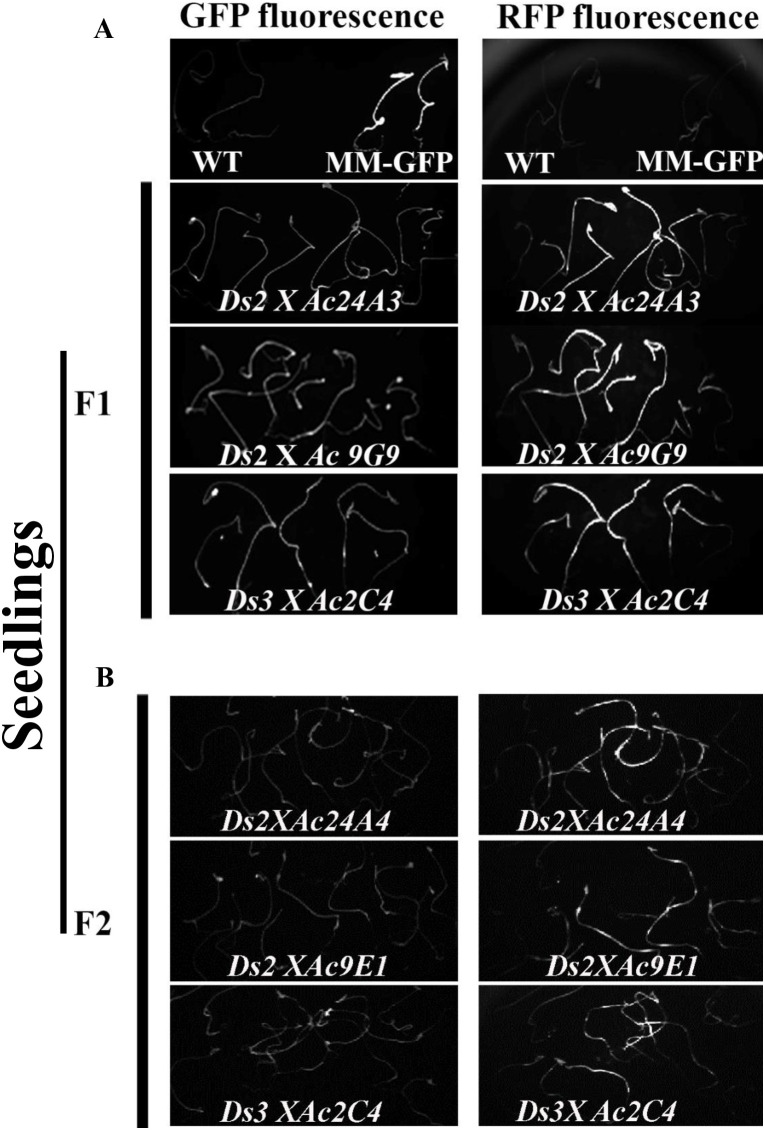
*In vivo* imaging of RFP and GFP fluorescence in F_1_ and F_2_ seedlings. A. Fluorescence imaging of F_1_ seedlings from the crosses *Ds-2 × Ac-24A3, Ds-2 × Ac-9G9*, and *Ds-3 × Ac-2C4,* captured under a GFP filter (left panel) and an RFP filter (right panel). Seedlings were illuminated using a UV lamp. B. Fluorescence Imaging of F_2_ seedlings from the crosses *Ds-2 × Ac-24A4, Ds-3 × Ac-2C4*, and *Ds-2 × Ac-9E1,* using the same setup with GFP (left panel) and RFP (right panel) filters.

In both sets of crosses, the presence of GFP and RFP fluorescence in F_1_ seedlings indicated successful transfer of the *Ds* transgene (♂) (*RFP*^*+*^*/GFP*^*+*^) to the *Ac* (♀) (*GFP*^*+*^) lines, as the maternal *Ac* (♀) lines lacked RFP (**Fig 3**). The presence of the *GFP* and *RFP* genes was further confirmed through PCR using transgene-specific primers. The F_1_ plants were allowed to self-pollinate, and the resulting F_2_ plants were subsequently screened for *Ds* mobilization.

Untransformed wild-type (WT) seedlings did not exhibit GFP or RFP fluorescence. The MM-GFP positive control lines displayed GFP fluorescence but no RFP fluorescence.

**Fig 4:**
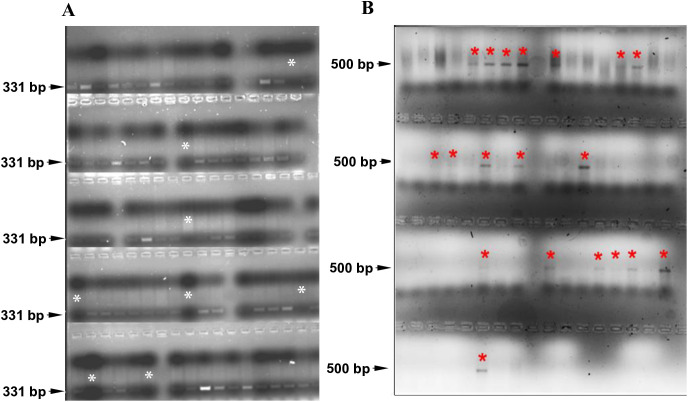
Analysis of *Ac-Ds* segregation in the F_2_ generation. A. PCR amplification using *GFP*-specific primers. A 331 bp amplicon indicates the presence of either both *Ac* and *Ds* elements or *Ac* alone. A white asterisk marks the absence of the GFP amplicon. B. PCR amplification using *RFP*-specific primers. A 500 bp amplicon signifies the presence of the mobilized *Ds* element only. A red asterisk*) denotes samples where only the RFP amplicon was detected.

For the F_2_ plants, screening was conducted to select GFP-negative (GFP^-^) and RFP-positive (RFP^+^) individuals.. Approximately 1,000 F_2_ seedlings from the second set of crosses were screened for *Ds* segregation. Out of 800 surviving F_2_ plants, 120 (15%) did not show amplification of the GFP gene by PCR, indicating the excision of the *Ds* element. These GFP^-^plants were then screened using RFP-specific primers (**Fig 4**). Among these, sixty plants (7.5%) showed amplification of the *RFP* sequence (~500 bp product), confirming the excision and reinsertion of the *Ds* elements (S8 Table). The genomic DNA from these *GFP*^*-*^*/RFP*^*+*^ plants was further analyzed to determine the location of the mobilized *Ds* elements.

A subset of F₂ progeny from crosses between *the Ds* transposon and the *Ac* donor lines exhibited distinct phenotypic variations compared to the wild-type Ailsa Craig (S9 Fig.). These included potato-like leaves with reduced serration and a leathery texture, and floral abnormalities such as altered anther cones. These morphological changes suggest underlying genetic disruptions caused by *Ds* insertion and provide promising candidates for further molecular analysis into tomato developmental pathways.

### Isolation of Flanking Sequences from RFP^+^ Lines

**Fig 5:**
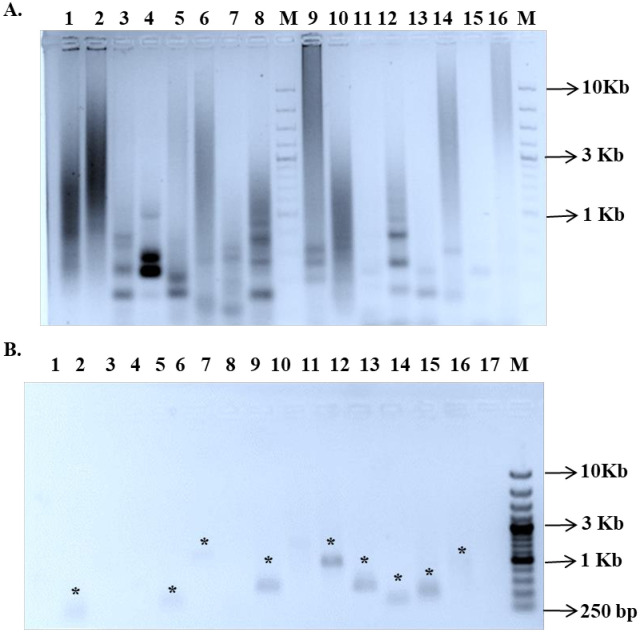
Inverse PCR Analysis of *Ds*-tagged DNA in GFP  ⁻ /RFP ⁺ *Ds* F_2_ lines. A. Inverse PCR products generated from DNA isolated from leaves of *Ds*-only plants. B. Gel-purified amplicons of flanking sequences obtained post-PCR. Note: Asterisks indicate the amplicons of flanking sequences in different samples. **M:** 1 Kb Marker. **Lanes 1-16,** *Ds*-only plants.

The first set of crosses was analyzed using FPNI-PCR, which yielded only ten transposition hits (S1 Dataset, #1–10). To enhance detection sensitivity, inverse PCR was employed to identify the insertion sites of the transposed element in *GFP*^*-*^*/RFP*^*+*^ plants from the second set of crosses. The amplified products were visualized using agarose gel electrophoresis, cloned into the pJET 2.0 vector, and sequenced (**Fig 5**). This approach led to the isolation, cloning, and sequencing of 46 flanking sequences from the second set of RFP^+^ plants (S1 Dataset, #11–56). The longest amplicon was 1452 bp, with an average length of 1110 bp.

**Fig 6:**
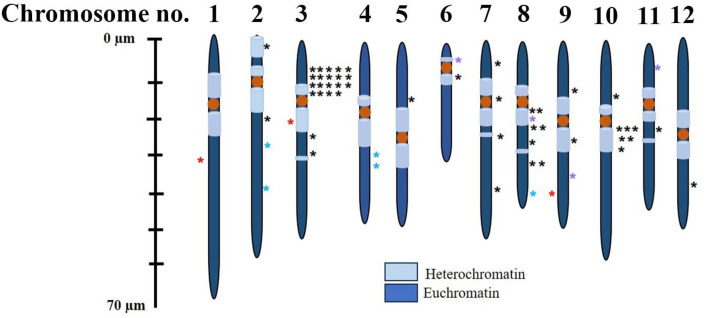
Schematic representation of *Ds* transposon distribution across the twelve tomato chromosomes. Transposon insertions were detected on 11 of the 12 chromosomes. Blue asterisks indicate insertions within genic regions, while black asterisks represent intergenic insertions. Purple asterisks denote insertions with unknown launch sites. Red asterisks on the left side of chromosomes 1 (*Ds-2*), 3 (*Ds-10-2*), and 9 (*Ds-8*) indicate the positions of known launch sites. The schematic incorporates data from both sets of crosses. Detailed genomic coordinates of the insertion sites are listed in S1 Dataset.

Sequence homology studies using a BLAST search against the tomato genome revealed that all 46 flanking sequences matched the tomato genome, with 24 unique insertion sites (52%). The highest number of insertions was found on chromosome 3, followed by chromosomes 8 and 10 (six insertions each), and chromosome 2 (four insertions). Chromosome 4 had two insertions, while the remaining chromosomes had one insertion each (S9 Table). No insertions were found on chromosome 1 (S1 Dataset, **Fig 6**).

Specifically, twenty-one insertions were mapped to chromosome 3, with nineteen occurring at the same chromosomal position. All three parental *Ds* lines (10−2, 10−8, and 10−1) shared an insertion at the identical locus on chromosome 3 (SL3.0ch03: 26,676,740–26,676,787), as they were derived from the same T_0_ line, *Ds-10*.

### Distribution of transposon insertions within intergenic and intragenic regions

A total of 8.7% of *Ds* insertions occurred within genic regions, including exons, introns, untranslated regions (UTRs), and within 1 Kb upstream or downstream of the gene. The remaining 91% of *Ds* insertions were located in intergenic regions. The insertion frequency in coding sequences (CDS) was 2.7% (S10 Fig.). Insertions in introns and promoters also accounted for 2.7% each. Analysis of genic insertions revealed disruptions in genes encoding several proteins, such as an ABC transporter family protein, DNA repair endonuclease, RNA-binding (RRM/RBD/RNP motifs) family protein, and ATP synthase α subunit genes. Additional insertions were found in intergenic regions near genes including those for a eukaryotic aspartyl protease family protein, BED zinc finger protein, cytochrome P450, phosphatidylinositol N-acetylglucosamine transferase subunit P-like protein, xanthine/uracil permease family protein, small nuclear ribonucleoprotein, 2-oxoglutarate (2OG) and Fe(II)-dependent oxygenase superfamily protein, and tetratricopeptide repeat (TPR)-like superfamily protein (S1 Dataset). Notably, nearly 40% of the flanking sequences had insertions upstream of an auxin efflux carrier family protein and downstream of a clade XVI lectin receptor kinase.

The relative positions of *Ds* insertions with respect to the nearest upstream and downstream genes, along with the approximate GC content of the insertion sites, were determined (S1 Dataset). GC content across these sites ranged from 30% to 100%. The five genic insertions exhibited high GC content. For instance, insertions on chromosome 2 in the RNA-binding protein and plastid transcriptionally active 14 protein had GC contents of 100% and 57%, respectively. On chromosome 4, insertions in the ATP synthase α-subunit and ABC transporter gene showed GC contents of 90% and 95.5%, respectively. An insertion in the DNA repair endonuclease XPF gene on chromosome 8 had a GC content of 85%.

## Discussion

### Independent *Ac* and *Ds* starter lines simplify large-scale mutant generation

Significant efforts have been made to develop functional genomics resources in tomato following the release of its complete genome sequence [29]. Most of these resources have been generated using EMS-mutagenized lines, with mutated genes identified through classical TILLING (Targeting Induced Local Lesions in Genomes) [30,31], NGS-based TILLING [32], and whole-genome sequencing approaches [33–35]. However, as EMS is a chemical mutagen that induces numerous random mutations across the genome, extensive backcrossing is required to validate gene function in individual mutants.

In contrast, T-DNA mutagenesis allows for the targeted insertion of T-DNA elements, which also serve as molecular tags to identify insertion sites and associate them with phenotypic changes. Despite these advantages, T-DNA mutagenesis is limited by its dependence on Agrobacterium-mediated transformation, which is not efficiently scalable in tomato due to the lack of a robust *in planta* transformation method. For example, **Pérez-Martín et al.** [36] used 22,700 explants to generate 7,842 transgenic tomato lines, of which 2,282 displayed tetraploidy. The high frequency of tetraploids complicates the use of T-DNA tagging for functional genomics, as each line must be screened for ploidy before analysis. Moreover, achieving genome-wide saturation with T-DNA insertions requires an extensive number of transformation events.

We employed maize *Activator/Dissociation* (Ac/Ds) transposons to generate gene knockouts through insertional mutagenesis. In our approach, tomato transformation is required only once that is to generate the *Ac* and *Ds* starter lines. A major challenge in the *Ac/Ds* transposition system is the identification of *Ds-only* lines, where the *Ds* element has excised and reinserted into a new genomic location. In rice, **Kolesnik et al.** [15] addressed this by first selecting GFP-negative plants and then applying the herbicide BASTA to identify resistant seedlings carrying transposed *Ds* elements. Similarly, our strategy involved a dual-screening process: first, selecting GFP-negative plants to eliminate *Ac* starter lines and untransposed *Ds* lines; second, identifying RFP-positive plants to confirm successful *Ds* transposition and determine the new insertion sites.

### *Ac* and *Ds* starter lines provide better resource than the classical approach

The reliability of selecting *Ds* transposition events at new genomic locations depends heavily on the expression levels of linked reporter genes. **Kolesnik et al.** [15] reported that nearly 20% of GFP-negative rice plants failed to exhibit BASTA resistance, highlighting limitations in marker-based selection. In plants, factors such as chromatin structure and the influence of neighboring genes within a 20 kb window can significantly affect transgene expression, either repressing or enhancing it [37]. In line with this, we observed considerable variability in GFP and RFP fluorescence intensity across different lines in our study. To overcome this variability and improve the accuracy of identifying genuine *Ds* transposition events, we incorporated PCR amplification of the reporter genes as a final confirmation step. The successful detection of new *Ds* insertion sites underscores the effectiveness of the two-component *Ac/Ds* transposon system in tomato, leveraging negative selection against the *Ac* transposase and untransposed *Ds* elements using GFP as a marker.

It is relevant to compare our study with previous efforts employing the *Ac/Ds* transposon system in tomato, particularly in the Micro-Tom [16] and M82 [17] cultivars. For example, **Meissner et al.** [16] developed 2,932 F_3_ families but were able to identify transposed loci for only 28 genes based on EST analysis. Their screening protocol for selecting F_2_ seedlings with germinally stable transpositions was notably more complex than ours. It involved multiple selection markers—acetolactate synthase resistance for excision, hygromycin or kanamycin resistance for reinsertion, and naphthalene acetamide resistance for transposition stabilization. Furthermore, F_1_ plants were first screened for chlorosulfuron resistance to identify lines carrying *Ds* insertions. The utility of their population, however, was further constrained by the unavailability of the tomato reference genome, which was not published until 2012. A further limitation of their approach stems from the use of Micro-Tom, a cultivar with a miniature phenotype resulting from hormonal and light-signaling mutations. These developmental anomalies can obscure the expression of agronomic traits and limit the applicability of findings to standard cultivars [38].

Using micropropagation, **Carter et al.** [17] generated 25 T_1_ lines from a single T_0_ line, resulting in 509 independent *Ds*-tagged lines. However, a major limitation of their approach was the lack of GFP expression due to the use of a monocot-specific promoter, which was ineffective in the dicotyledonous tomato. As a result, transgenic plant screening was restricted to a single hygromycin painting assay. In contrast, our starter lines, developed in a commercial tomato cultivar, overcome the limitations associated with Micro-Tom. Unlike the study by **Carter et al.** [17], the GFP and RFP reporters in our constructs are driven by dicot-specific promoters, enabling more effective visualization and selection. The use of independent *Ac* and *Ds* starter lines in our study provides a lasting resource to generate transposon-tagged lines, which can be screened by PCR and also fluorescence visualization. Importantly, our approach also minimizes dependence on tissue culture to generate transformants, as tissue culture is only limited generating the starter lines. The use of modified *Ac/Ds* constructs tailored for tomato enhances the utility of our lines as a robust platform for generating transposon-tagged mutants to investigate gene function and traits relevant to crop performance.

### Both linked and unlinked transpositions are observed in mutant progeny

A key advantage of using a transposase-expressing line in combination with a limited number of transposon-bearing lines as starter lines is the potential to generate a large number of mutant lines through genetic crossing. For effective genome-wide mutagenesis, it is crucial that *Ds* elements mobilize to diverse genomic locations, including sites distant from their original insertion. In tomato, *Ds* transposition has been reported to result in both linked and unlinked insertions [39,40], in contrast to monocots, where *Ds* insertions are typically closely linked to their launch sites [41]. Consistent with these findings, our study identified both linked and unlinked transpositions relative to the *Ds-10–2* launch site on chromosome 3, further underscoring the distinct transposition behavior of *Ds* elements in dicots compared to monocots.

An intriguing observation in our study was the occurrence of multiple *Ds* insertions at the same locus on chromosome 3. In *Arabidopsis*, **Parinov et al.** [12] reported transposition hot spots near the nucleolus organizer regions NOR2 and NOR4 using the *Ac/Ds* system. Similarly, in rice, *Ds* elements exhibited preferential transposition to a hot spot on chromosome 7 [15]. In tomato, previous studies have also reported a tendency for *Ds* elements to transpose within the same chromosome as the launch site [41]. In this context, the 19 insertions identified at a single site in our study may indicate the presence of a transposition hot spot. Alternatively, these plants could be progeny from a single F_1_ individual in which transposition occurred prior to gametogenesis, resulting in multiple siblings carrying the same insertion. However, further investigation is required to confirm this scenario. A more plausible explanation is that the *Ds-10–2* launch site lies proximal to the repeated insertion site, leading to frequent local transpositions. Such clustered insertions may result from higher chromatin accessibility near the launch site, facilitating repeated *Ds* integration in that genomic region.

### Transposon mutagenesis is random across the tomato genome

With the exception of chromosome 1, *Ds* elements were widely distributed across the remaining ten tomato chromosomes, suggesting numerous unlinked transpositions. However, as we only examined RFP-positive lines, the true number of unlinked transpositions may be higher. It is possible that we missed F_2_ lines where *Ds* elements transposed to new locations but failed to segregate from the empty *Ds* launch pad or the *Ac* transposase-bearing GFP. Despite this, the observed distribution of linked and unlinked transpositions in our study aligns with previous findings, which show that, unlike monocots, both linked and unlinked transpositions occur at nearly equal frequencies in tomato [39]. Importantly, the absence of specific hotspots for unlinked transpositions suggests that even a small number of *Ds* starter lines and a single *Ac* transposase line could lead to extensive, genome-wide *Ds* insertions in tomato.

The distribution of linked versus unlinked transpositions is thought to be influenced by the developmental stage at which transposition occurs in the F_1_ plant. Transpositions that take place during early plant development are more likely to result in linked transpositions, while those initiated during reproductive development tend to lead to unlinked transpositions in progeny derived from a single F_1_ plant. While linked transpositions are typically associated with *Ac* transposase driven by a heterologous promoter, such as the 35S promoter [42], unlinked transpositions occur when *Ac* transposase is driven by a host plant promoter [43]. Given that we analyzed F_2_ plants from a pooled population of F_1_ plants, distinguishing the precise timing of transposition is challenging. However, our observation of both linked and unlinked transpositions using *Ac* transposase driven by the 35S promoter suggests that this promoter does not hinder the occurrence of unlinked transpositions in tomato.

Theoretically, *Ds* transpositions are expected to occur randomly across the genome, without a preference for specific sites. However, transposition frequencies tend to be lower in heterochromatic regions compared to euchromatin regions [44,45]. In our analyzed population, the excision and reinsertion frequencies of the *Ds* element were 15% and approximately 7.5%, respectively. Notably, several *Ds* insertions were found in heterochromatic regions, suggesting no inherent bias toward gene-rich regions. Furthermore, no preference for GC-rich regions was observed, as the transpositions were distributed across a wide range of GC content.

The genic regions in tomatoes account for only about 8.1% of the genome [46] The 8.7% of *Ds* insertions occurred in genic regions is consistent with the proportion of genic region in tomato., and. Many of the intergenic insertions were located near genes with essential functions. While genic mutations are typically responsible for mutant phenotypes, emerging evidence suggests that mutations in intergenic regions, promoters, UTRs, and introns can also influence phenotypes by modulating gene expression [31]. In tomato, an intergenic Ds insertion near Solyc12g010240.2.1 and Solyc01g010250.2.1 resulted in a dominant phenotype, while an intronic insertion in Solyc12g006160.1.1 upregulated a distal WRKY gene [17]. Transposon insertions in promoter regions affected the expression of nearby genes, resulting in altered floral development and stress responses in *Arabidopsis* [47]. Similarly, *Ds* insertions in intergenic regions induced defects in male gametophyte development and function [48]. Ostensibly, intergenic insertions can indirectly influence gene function by altering gene regulation, supporting their value in functional genomics.

### Limitations and challenges of the *Ac/Ds* system

Insertional mutagenesis using *Ac*/*Ds* transposable elements remains a useful tool for functional genomics in tomato. In this study, 8.7% of insertions occurred within annotated genes, which is close to tomato genic regions comprising about 8.1% of the genome [46]. Though most insertions were in intergenic regions, these may also contribute to phenotype by influencing LncRNA, the promoter, and enhancer elements [49]. The absence of insertions on chromosome 1 is likely due to the limited number of analysed lines rather than intrinsic chromosomal bias. Larger mutant populations and diverse starter lines should enable insertions on all chromosomes, as shown in larger-scale mutagenesis studies [15].

The prevalence of insertion site bias may hamper the potential of the *Ac*/*Ds* system for genome-wide generation of mutants. Major factors contributing to this bias include “local hopping” (preferential insertion near the original site), chromatin structure, DNA methylation, and target site sequence preferences—especially relevant for tomato’s large and complex genome [50]. To improve genome coverage, combining *Ac/Ds* mutagenesis with an alternate approach such as CRISPR/Cas9 is ideal. Additionally, engineering modified transposon systems or employing inducible/tissue-specific strategies may enhance insertion diversity [51].

### Genome editing versus transposon mutagenesis

As genome editing emerges as a powerful tool for studying gene function, it is useful to compare it with insertion mutagenesis. Unlike insertion mutagenesis, genome editing typically requires the transformation of multiple gRNA constructs designed to disrupt the function of specific genes in plants. Genome editing primarily results in deletions, due to the nature of the editing tools. For example, in maize, most genome-edited genes exhibited deletions (60%) rather than insertions (32.5%) [52], a pattern similar to fast-neutron mutagenesis, which predominantly generates insertions and deletions [53]. In contrast, *Ds*-induced mutations mainly lead to gene knockouts and can also be used for activation tagging. Furthermore, once a set of starter lines is established, insertional mutagenesis can be scaled up without the complexity of designing gRNAs or transforming plants.

## Conclusion

In summary, we present a robust two-component *Ac/Ds* system for insertional mutagenesis in tomato. Our starter lines are available for distribution to researchers interested in generating tomato insertional mutant lines for various studies or expanding the mutant resources by developing additional lines. By sharing these lines across different research groups, a community-wide effort can be launched to saturate the tomato genome with insertional mutants. This collaborative approach will significantly enhance our understanding of gene functions and enable comprehensive studies of tomato genetics and biology.

## Supporting information

S1 Fig A schematic representation of the steps involved in mobilizing the *Ac-Tpase (pSSZ36)* construct into the *pBINPLUS* plasmid. (PDF)

S2 Fig Generation of Ac-TPase constructs through various molecular steps. (PDF)

S3 Fig A schematic representation of the steps involved in mobilizing the *Ds (pSQ3)* construct into the *pBINPLUS* plasmid. (PDF)

S4 Fig Generation of *Ds* construct. (PDF)

S5 Fig Detection of the transgene in *Ac*-*TPase* and *Ds* T_0_ plants by PCR. (PDF)

S6 Fig Southern blots of *Ac-TPase* lines for ascertaining transgene presence and copy number. (PDF)

S7 Fig Screening process for selecting plants with *Ds* transposition. (PDF)

S8 Fig Kanamycin painting assay for the identification of transgenic plants. (PDF)

S9 Fig Phenotypic alterations in F_2_ progeny of *Ac* X *Ds* crosses compared to Arka Vikas (WT). (PDF)

S10 Fig Distribution of *Ds* insertion sites in intergenic and intragenic regions. (PDF)

S1 Table Composition of MS media used in various transformations and regeneration steps. (PDF)

S2 Table Genetic transformation efficiency of *Ac-Tpase* (A) and *Ds* starter lines (B). (PDF)

S3 Table The sequences of primers used for PCR. (PDF)

S4 Table The sequences of primers used in FPNI-PCR. (PDF)

S5 Table The sequences of primers used for inverse PCR. (PDF)

S6 Table Kanamycin resistance assay of *Ac-TPase* starter lines. (PDF)

S7 Table The chromosomal location of the *Ds* launching pad. (PDF)

S8 Table Step-wise seedlings screened for *Ds* transposition. (PDF)

S9 Table Distribution of *Ds*-tagged lines on different chromosomes of tomato. (PDF)

S1 Dataset Location of *Ds* transposition in the tomato genome. (XLSX)

S1 File Raw images for gel/blots. (PDF)
