# Supplementary material for: Development of Ac- and Ds-tagged starter lines for large-scale transposon-mutagenesis in tomato
Source: PLoS One. 2025 Nov 19;20(11):e0335612. doi: 10.1371/journal.pone.0335612 (PMC12629433; doi:10.1371/journal.pone.0335612)
Supplement: S1 Fig — (PDF) [file pone.0335612.s001.pdf]

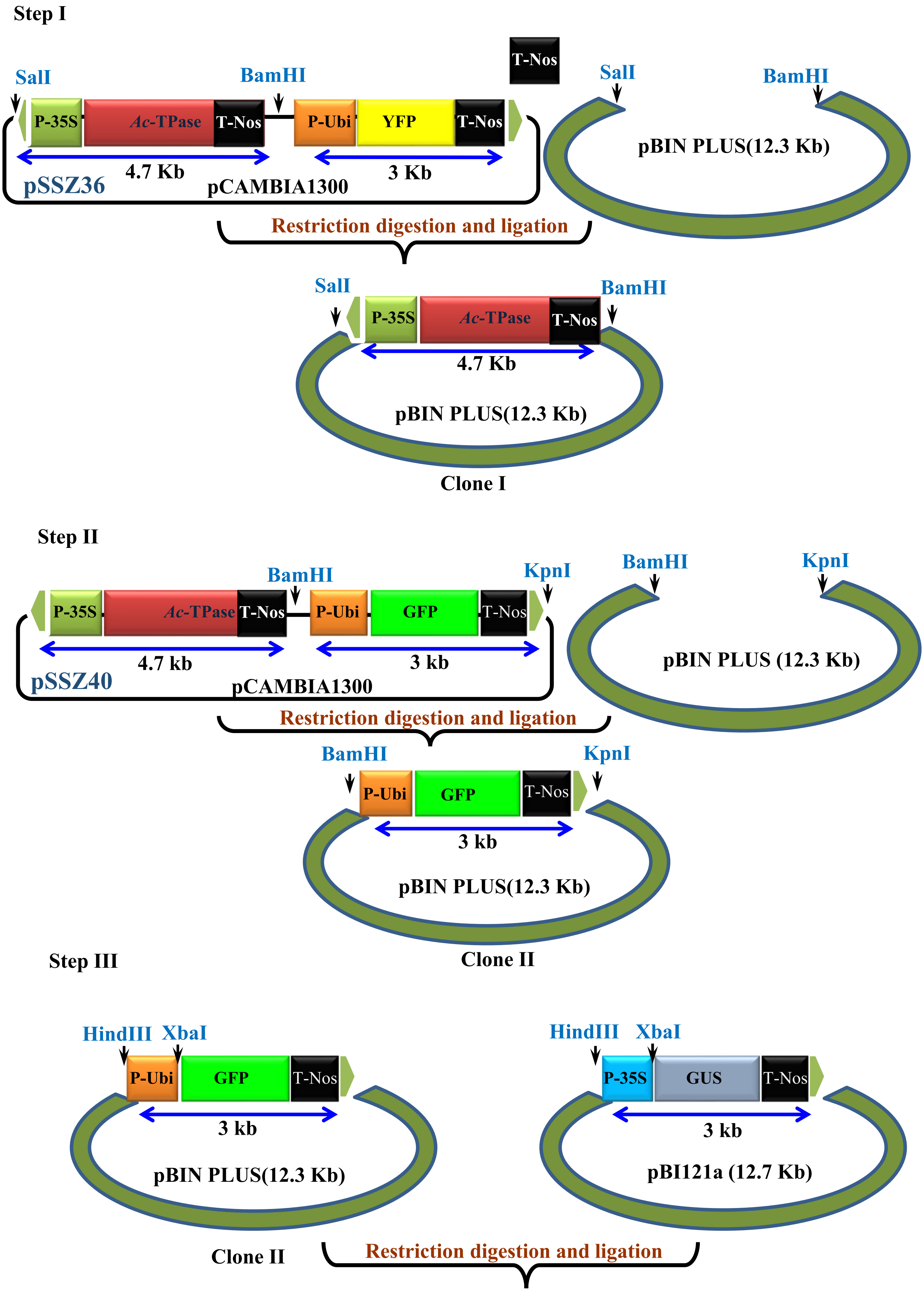
Step I
SalI
BamHI
T-Nos
SalI
BamHI
P-35S
Ac-TPase
T-Nos
P-Ubi
YFP
T-Nos
pBIN PLUS(12.3 Kb)
4.7 Kb
3 Kb
pSSZ36
pCAMBIA1300
Restriction digestion and ligation
SalI
BamHI
P-35S
Ac-TPase
T-Nos
4.7 Kb
pBIN PLUS(12.3 Kb)
Clone I
Step II
BamHI
KpnI
BamHI
KpnI
P-35S
Ac-TPase
T-Nos
P-Ubi
GFP
T-Nos
pBIN PLUS (12.3 Kb)
4.7 kb
3 kb
pSSZ40
pCAMBIA1300
Restriction digestion and ligation
BamHI
KpnI
P-Ubi
GFP
T-Nos
3 kb
pBIN PLUS(12.3 Kb)
Clone II
Step III
HindIII
XbaI
P-Ubi
GFP
T-Nos
3 kb
pBIN PLUS(12.3 Kb)
Clone II
HindIII
XbaI
P-35S
GUS
T-Nos
3 kb
pBI121a (12.7 Kb)
Restriction digestion and ligation

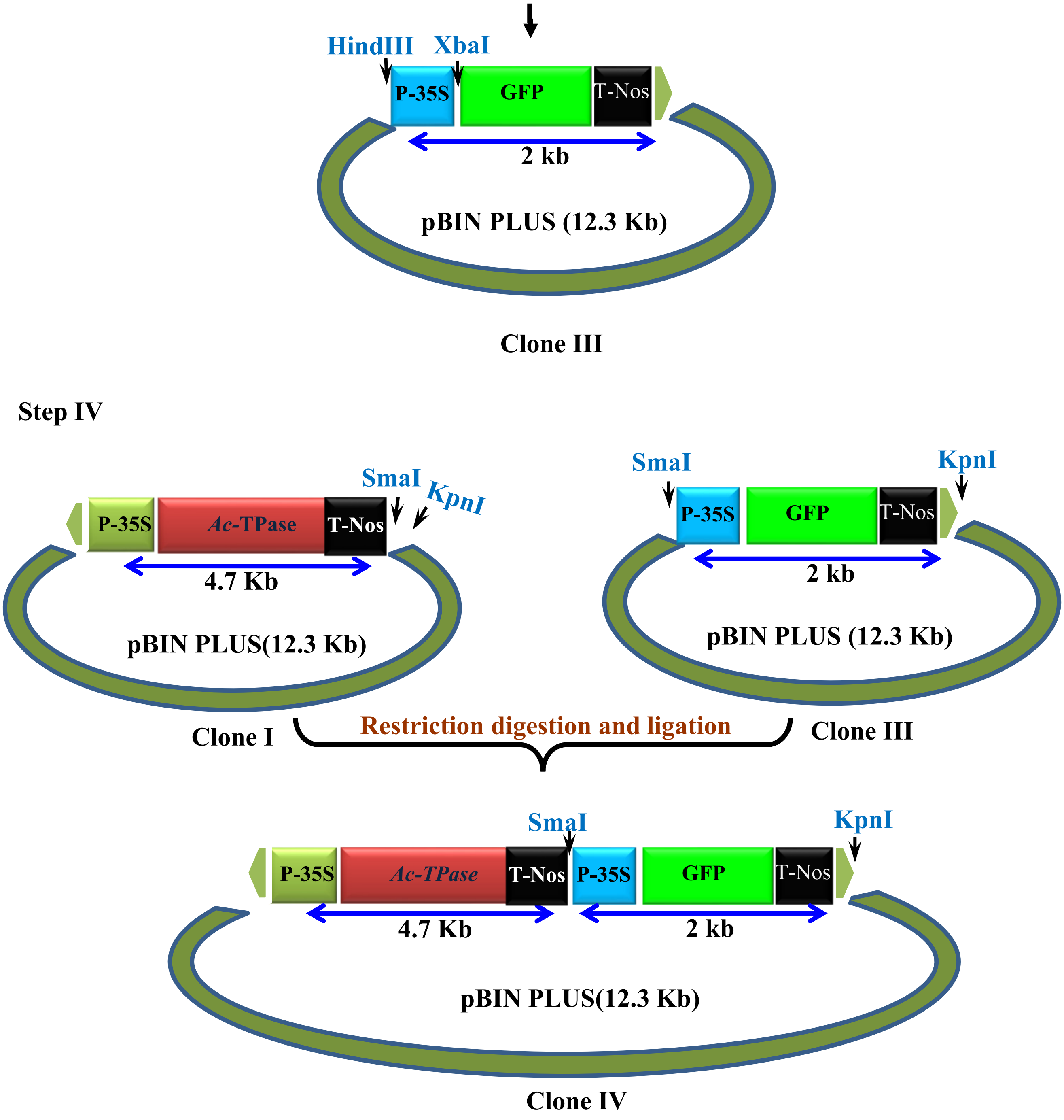


**S1 Fig.** A schematic representation of the steps involved in mobilizing the *Ac-Tpase (pSSZ36)* construct into the *pBINPLUS* plasmid. The scheme also illustrates the replacement of the maize ubiquitin promoter with the *35S* promoter to drive *GFP* expression (*pSSZ40*), followed by the final mobilization of the modified construct into the *pBINPLUS* plasmid alongside the *Ac-TPase* construct.
