# Supplementary material for: Development of Ac- and Ds-tagged starter lines for large-scale transposon-mutagenesis in tomato
Source: PLoS One. 2025 Nov 19;20(11):e0335612. doi: 10.1371/journal.pone.0335612 (PMC12629433; doi:10.1371/journal.pone.0335612)
Supplement: S2 Fig — (PDF) [file pone.0335612.s002.pdf]

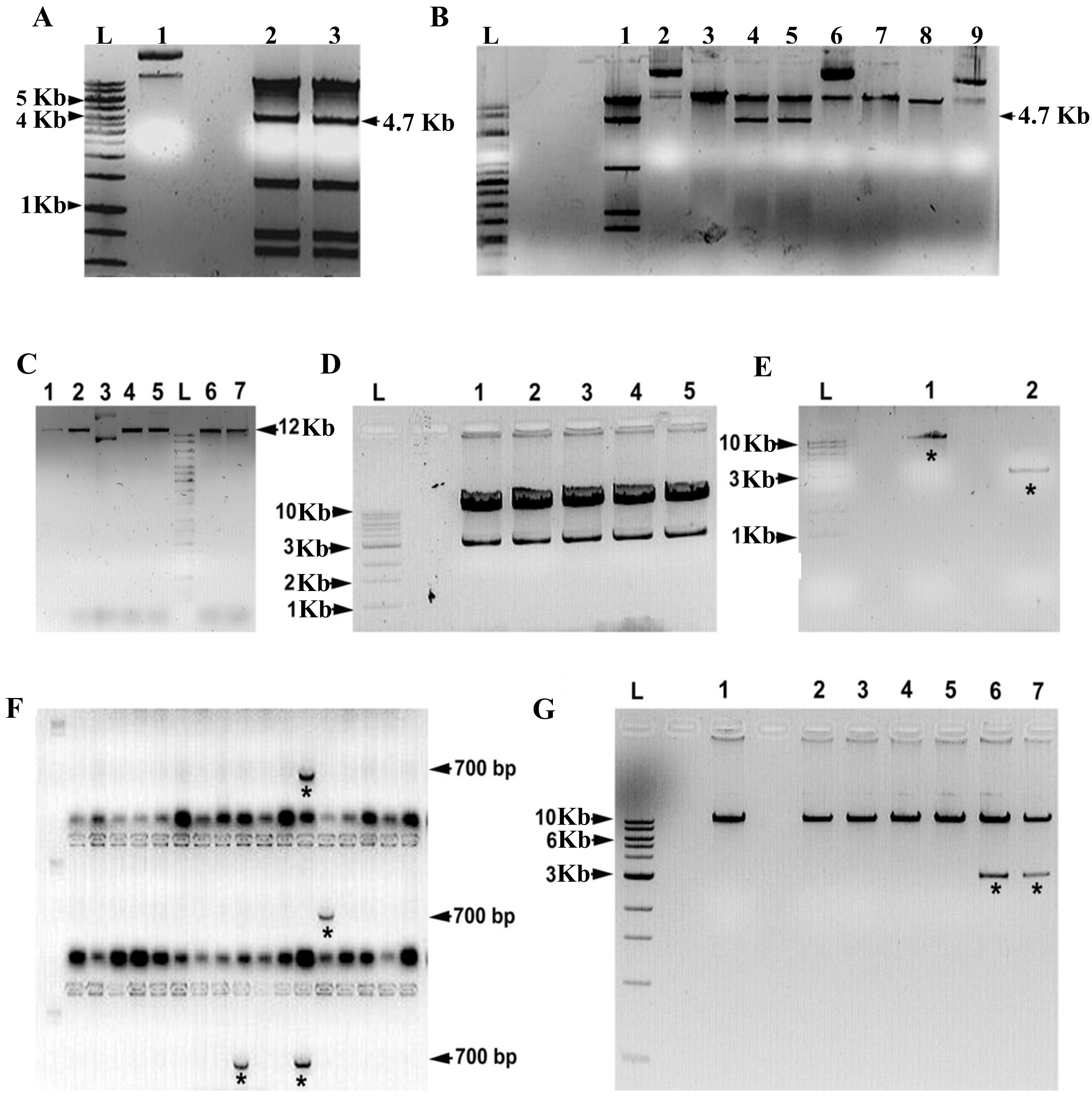
A
L 1 2 3
5 Kb
4 Kb
1Kb
4.7 Kb
B
L 1 2 3 4 5 6 7 8 9
4.7 Kb
C
1 2 3 4 5 L 6 7
12Kb
D
L 1 2 3 4 5
10Kb
3Kb
2Kb
1Kb
E
L 1 2
10Kb
3Kb
1Kb
*
*
F
700 bp
*
700 bp
*
700 bp
* *
G
L 1 2 3 4 5 6 7
10Kb
6Kb
3Kb
* *

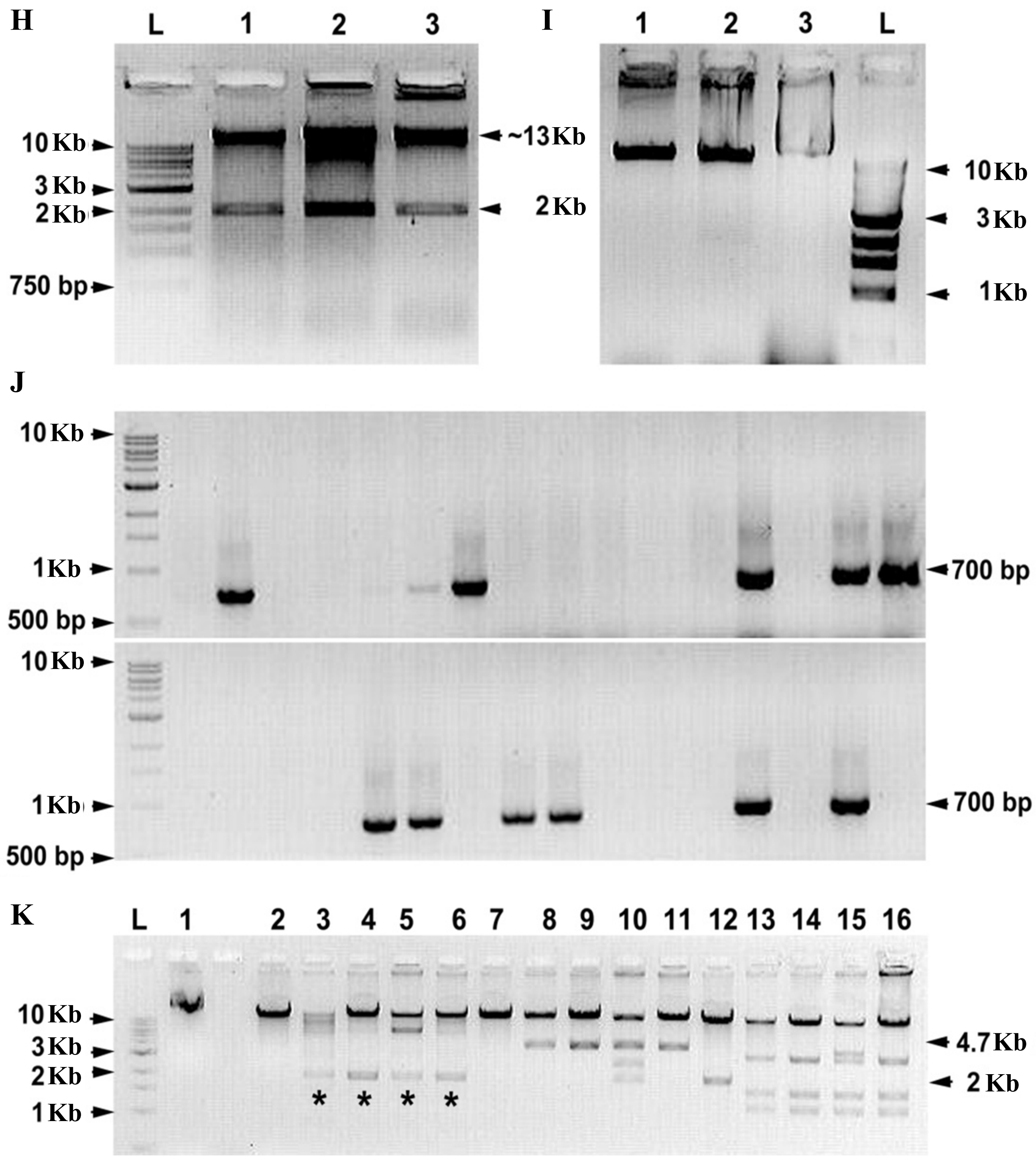
H
L 1 2 3
10 Kb
3 Kb
2 Kb
750 bp
~13 Kb
2 Kb
I
1 2 3 L
10 Kb
3 Kb
1 Kb
J
10 Kb
1 Kb
500 bp
700 bp
10 Kb
1 Kb
500 bp
700 bp
K
L 1 2 3 4 5 6 7 8 9 10 11 12 13 14 15 16
10 Kb
3 Kb
2 Kb
1 Kb
* * * *
4.7 Kb
2 Kb

S2 Fig. Generation of the Ac-TPase constructs through various molecular steps.

A. Restriction digestion of the *pSSZ36* vector. Lane 1, Undigested plasmid; Lane 2-3, Plasmid digested with BamHI/SalI.

B. Restriction digestion of recombinant *pBINPLUS* plasmid and pSSZ36 bearing plasmid. Lane 1, *pSSZ36* plasmid digested with BamHI/SalI showing the release of 4.7 Kb band and three additional bands; Lane 2, Undigested *pSSZ36* plasmid; Lane 3, Recombinant *pBINPLUS* plasmid digested with BamHI; Lane 4-5, Release of 4.7 Kb fragment from recombinant *pBINPLUS* plasmid digested with BamHI/SalI (**Note**: Only one 4.7 Kb band is released in compared to *pSSZ36* that shows four bands along with 4.7 kb band); Lane 6, Undigested recombinant *pBINPLUS* plasmid; Lane 7-8, *pBINPLUS* digested with BamHI/SalI; Lane 9, Undigested *pBINPLUS* plasmid.

C. Restriction digestion profile of *pBINPLUS*; Lane 1-2, Plasmid digested with BamHI; Lane 3, Undigested plasmid; Lane 4-5, Plasmid digested with KpnI; Lane 6, Plasmid digested sequentially with BamHI followed by KpnI; Lane 7, Plasmid digested sequentially with KpnI followed by BamHI.

D. Lane 1-5, *pSSZ40* vector digested with BamHI/KpnI.

E. Gel eluted plasmids digested with BamHI/KpnI; Lane 1, Linearized *pBINPLUS* plasmid; Lane 2, 3 Kb cassette from pSSZ40 plasmid.

F. Colony PCR using *GFP*-specific primers; **Note,** A few colonies showed the presence of a 700 bp product.

G. Restriction digestion of plasmid (*pBINPLUS* having *ZmUbi+sGFP+NOSter* cassette) isolated from PCR positive colonies; Lane 1-7, transformed *pBINPLUS* plasmid digested with BamHI/KpnI. Lanes 6-7 show the release of a 3 Kb *GFP* reporter cassette. *Mark in each gel shows the amplified PCR product or release of the desired insert.

H. Release of *GFP* reporter gene cassette from *pBINPLUS* upon digestion with SmaI and KpnI; Lane 1-3 show release of 2 Kb reporter gene cassette.

I. Restriction profile of *pBINPLUS* having *Ac-TPase* element. Lane 1-2, Linearized plasmid after digestion with SmaI and KpnI; Lane 3, Undigested plasmid. (**Note**: Linearized plasmid in Lane 1-2 has more gel mobility than undigested plasmid).

J. Colony PCR using *GFP-specific* primers. The presence of insert was identified by PCR amplification of a 700 bp product. **Note**: Few colonies show the presence of a 700 bp product.

K. Restriction profile of cloned (*sGFP* reporter gene cassette with *Ac-TPase* element in *pBINPLUS* plasmid); Lane 1, Undigested plasmid; Lane 2, Linearized *pBINPLUS* with *Ac-TPase* element after digestion with SmaI and KpnI; Lane 3-6, Release of 2 Kb *GFP* reporter cassette after digestion with SmaI and KpnI (marked with*); Lane 7, Linearized *pBINPLUS* with *GFP* reporter cassette after digestion with BamHI/SalI; Lane 8-11, Release of 4.7 Kb *Ac-TPase* after digestion with BamHI/SalI; Lane 12, Digestion of *pBINPLUS* plasmid with *GFP* reporter cassette with HindIII; Lane 13-16, Recombinant clones digested with HindIII enzymes **Note**: *Ac-TPase* has five internal restriction sites for HindIII enzymes. In each gel, **Lane L** 1 Kb DNA ladder.
