# Supplementary material for: Development of Ac- and Ds-tagged starter lines for large-scale transposon-mutagenesis in tomato
Source: PLoS One. 2025 Nov 19;20(11):e0335612. doi: 10.1371/journal.pone.0335612 (PMC12629433; doi:10.1371/journal.pone.0335612)
Supplement: S3 Fig — (PDF) [file pone.0335612.s003.pdf]

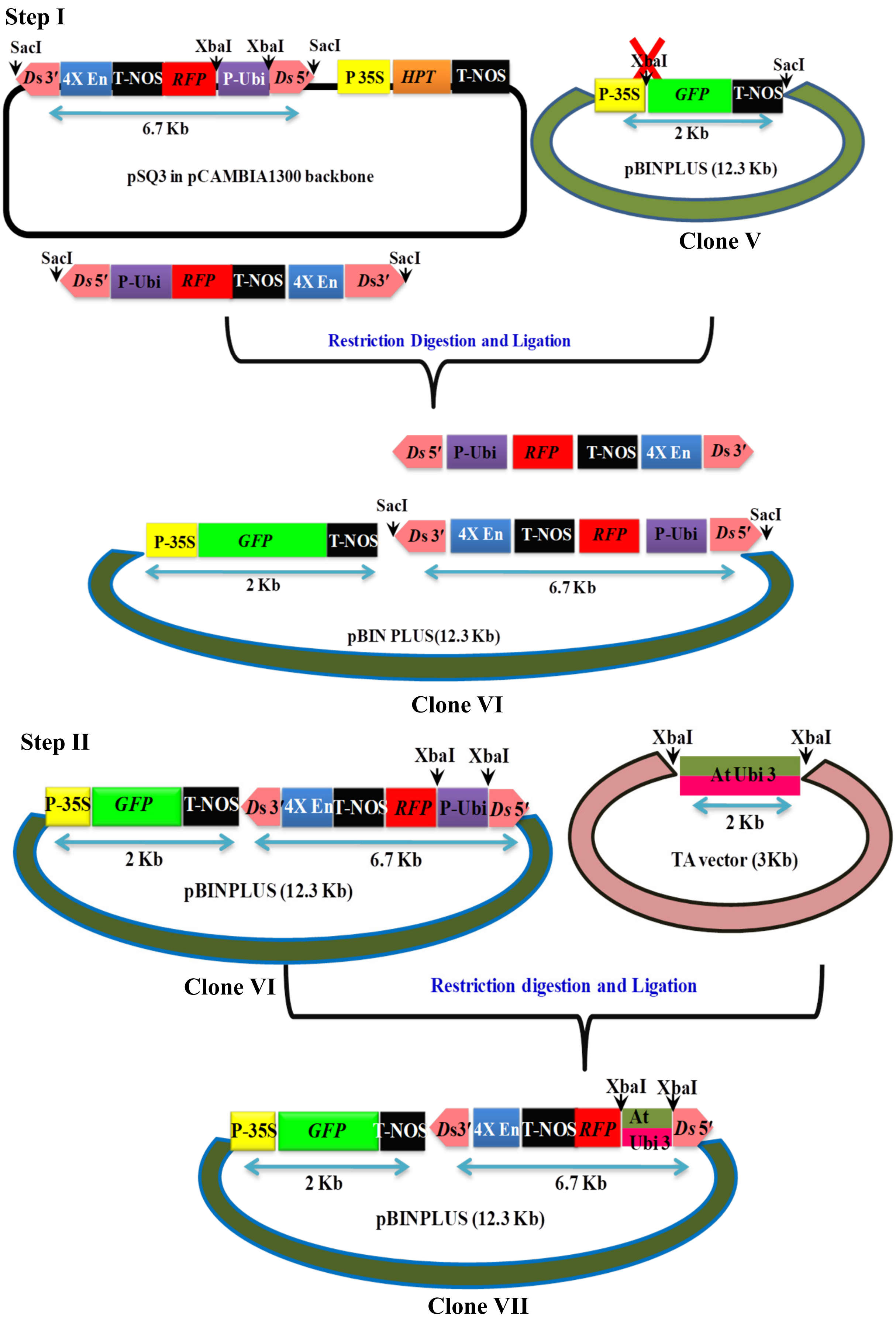

S3 Fig. A schematic representation of the steps involved in mobilizing the *Ds (pSQ3)* construct into the *pBINPLUS* plasmid. The scheme illustrates the replacement of the maize ubiquitin promoter with the Arabidopsis ubiquitin promoter to drive *Ds* expression, and the replacement of the hygromycin gene with GFP, and the mobilization of the modified construct into the *pBINPLUS* plasmid.
