# Supplementary material for: Development of Ac- and Ds-tagged starter lines for large-scale transposon-mutagenesis in tomato
Source: PLoS One. 2025 Nov 19;20(11):e0335612. doi: 10.1371/journal.pone.0335612 (PMC12629433; doi:10.1371/journal.pone.0335612)
Supplement: S4 Fig — (PDF) [file pone.0335612.s004.pdf]

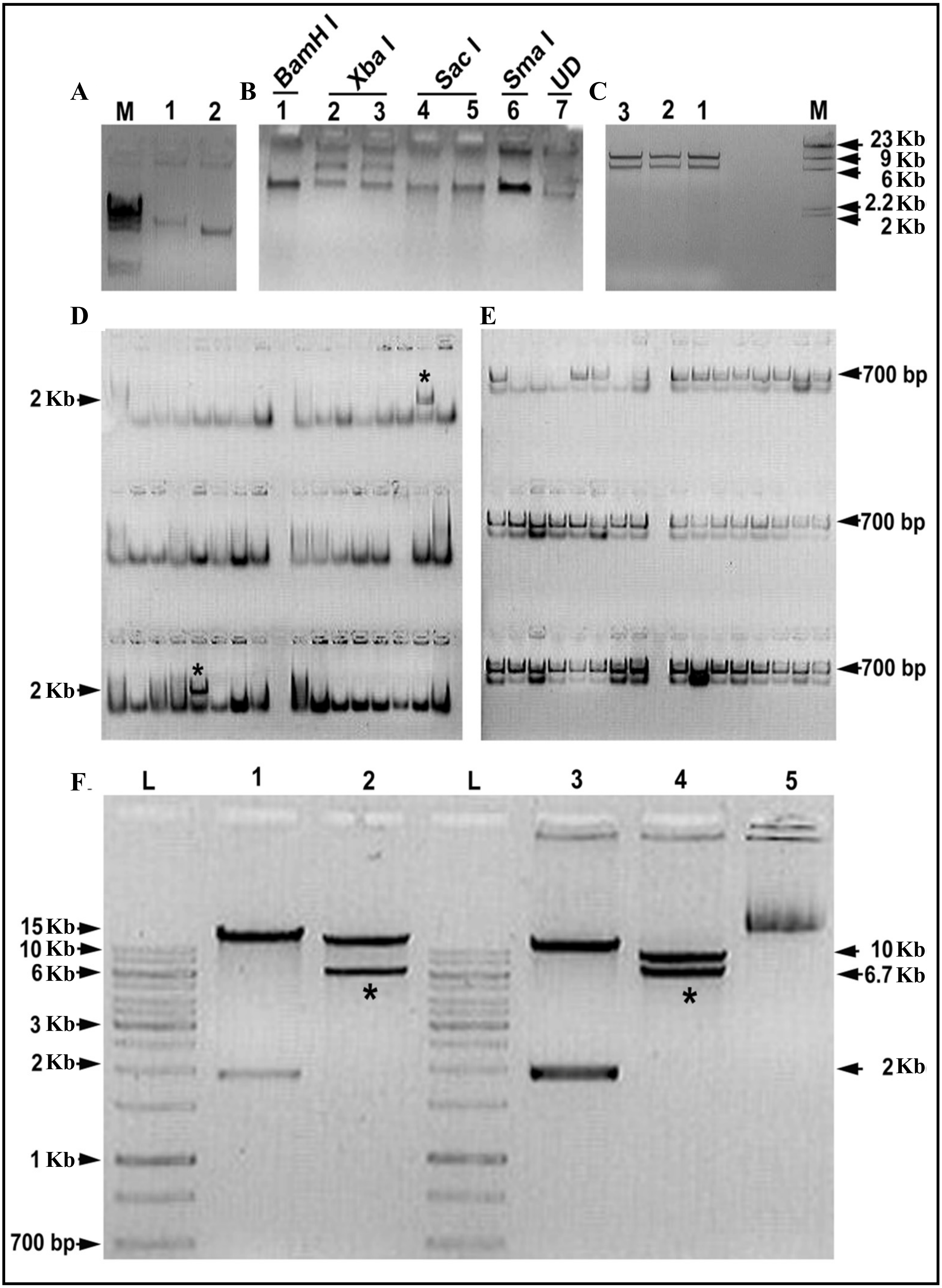
A
M 1 2
B
BamH I
Xba I
Sac I
Sma I
UD
1 2 3 4 5 6 7
C
3 2 1 M
23 Kb
9 Kb
6 Kb
2.2 Kb
2 Kb
D
2 Kb
*
*
2 Kb
E
700 bp
700 bp
700 bp
F
L 1 2 L 3 4 5
15 Kb
10 Kb
6 Kb
3 Kb
2 Kb
1 Kb
700 bp
*
*
10 Kb
6.7 Kb
2 Kb

S4 Fig. Generation of *Ds* construct.

A. Linearization of *pBINPLUS* plasmid harboring *GFP* reporter cassette with XbaI. Lane 1, Plasmid digested with XbaI; Lane 2, Undigested plasmid; Lane M, λ DNA/ HindIII digested ladder.

B. Confirmation of disruption of the XbaI restriction site in the pBINPLUS plasmid harboring the *GFP* reporter cassette. Lane 1, Linearized plasmid after digestion with BamHI enzyme; Lane 2-3, Undigested plasmid after digestion with XbaI enzyme; Lane 4-5, Linearized plasmid after digestion with SacI enzyme; Lane 6, Linearized plasmid after digestion with SmaI enzyme; Lane 7, Undigested plasmid.

C. Release of *Ds* cassette from pSQ3 plasmid; Lane 1-3, 6.7 Kb fragment released from pSQ3 after digestion with the SacI enzyme; Lane M**,** λ DNA/ HindIII digested ladder.

D. Colony PCR of recombinant colonies with *ZmUbi* promoter-specific primers. **Note:** Amplicon of a 2 Kb size (*) indicates the presence of insert.

E. Colony PCR with *GFP*-specific primers. **Note:** Amplicon of 700 bp size (*) indicates the presence of backbone.

F. Mobilization of 6.7 Kb *Ds* element cassette to pBINPLUS binary vector with *GFP* reporter gene. Lane 1, Release of 2 Kb *ZmUbi* promoter from pBINPLUS plasmid after digestion with XbaI; Lane 2, Release of 6.7 Kb *Ds* cassette from pBINPLUS after digestion with SacI; Lane 3, Release of 2 Kb *ZmUbi* promoter from pSQ3 plasmid after digestion with XbaI; Lane 4, pSQ3 plasmid digested with SacI releases a 6.7 Kb fragment along with 10 Kb backbone; Lane 5, Undigested pSQ3 plasmid; **Lane L,** 1 Kb DNA ladder in each gel.
