# Supplementary material for: Development of Ac- and Ds-tagged starter lines for large-scale transposon-mutagenesis in tomato
Source: PLoS One. 2025 Nov 19;20(11):e0335612. doi: 10.1371/journal.pone.0335612 (PMC12629433; doi:10.1371/journal.pone.0335612)
Supplement: S5 Fig — (PDF) [file pone.0335612.s005.pdf]

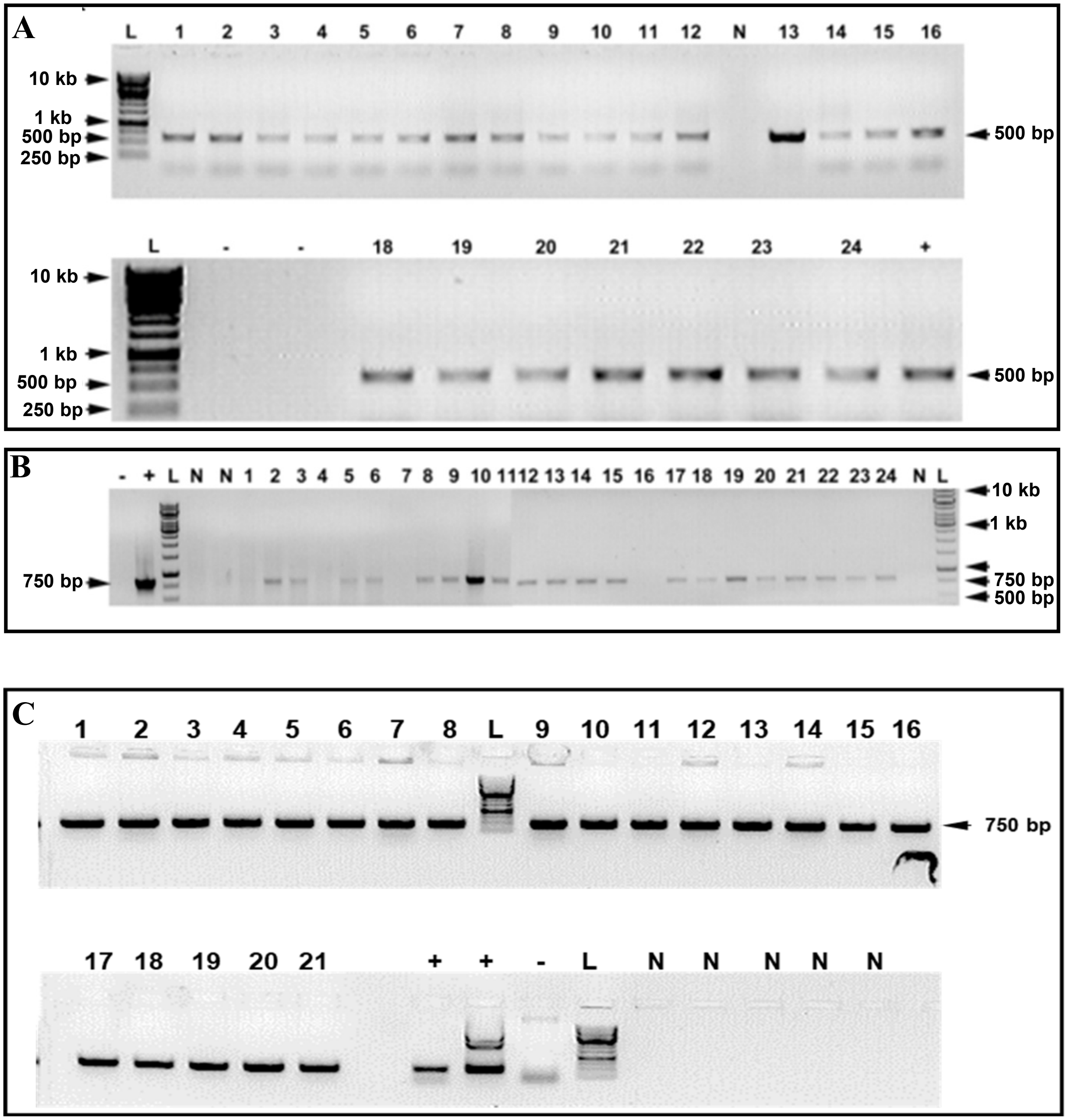

S5 Fig. Detection of the transgene in *Ac-TPase* and *Ds* T_0 plants by PCR. **A**. PCR with primers specific to *Ac-TPase* shows amplification of ~500 bp product of transgene. **B-C**. PCR with primers specific to *NPTII* shows amplification of ~750 bp product in *Ac-TPase* and *Ds* plants, respectively. In each gel, Lane L signifies a 1 Kb DNA ladder. Symbols + and – designate positive (+) and negative control (**-**). Plasmid DNA of the construct employed in the transformation was used as a positive control, and the genomic DNA of the untransformed plant was used as a negative control. **N** designates an empty lane. Numbers on each lane indicate the line number of the respective T_0 plant.
