# Supplementary material for: Development of Ac- and Ds-tagged starter lines for large-scale transposon-mutagenesis in tomato
Source: PLoS One. 2025 Nov 19;20(11):e0335612. doi: 10.1371/journal.pone.0335612 (PMC12629433; doi:10.1371/journal.pone.0335612)
Supplement: S6 Fig — (PDF) [file pone.0335612.s006.pdf]

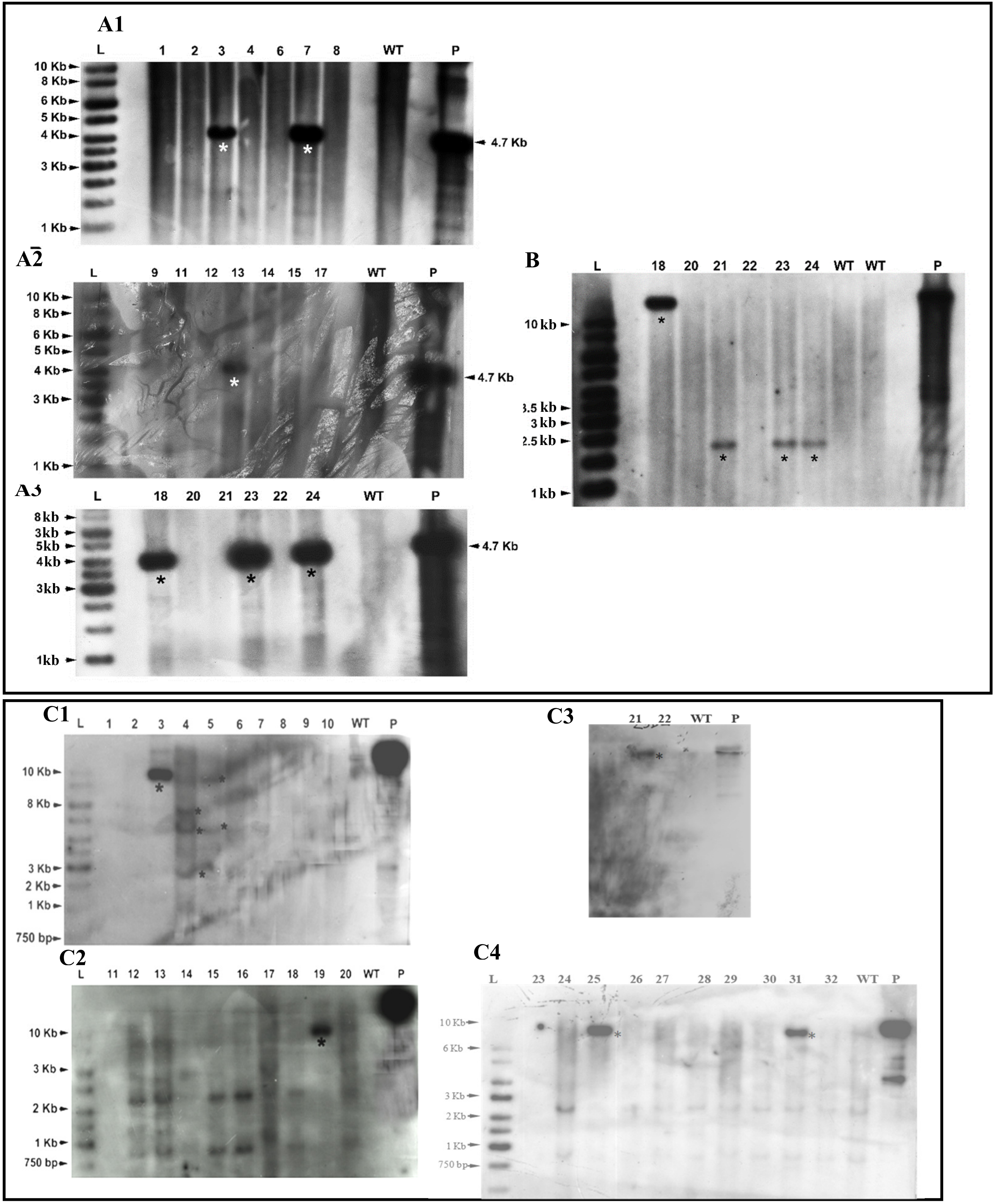

S6 Fig. Southern blots of *Ac-TPase* lines for ascertaining the transgene presence and copy number. **A1-A3**. For monitoring the transgene integration in transformed lines, a 4.7 kb (*Ac-TPase* gene) fragment was used as a probe. The genomic DNA of T_0 plants was digested with BamHI/SalI. **B.** For checking the copy number of the transgene. The genomic DNA of *Ac-TPase* T_0 plants was digested with BamHI and probed with the radiolabelled *NPTII* gene. **C1-C4.** Genomic DNA of *Ac-TPase* T_2 plants digested with BamHI. The blot was probed with a radiolabelled *NPTII* gene. Lane **L**, 1 kb DNA Ladder; P, Plasmid DNA of the construct used for transformation; WT, Wild type genomic DNA (negative control). **Note:** Lanes showing the presence of transgene in the Transgenic lines are marked with a star (*) symbol. The numbers on top of each lane indicate the line number of the respective T_0 /T_2 plant. **Note:** Star mark (*) indicates the presence of *Ac-TPase* transgene in transgenic lines.
