# Supplementary material for: Development of Ac- and Ds-tagged starter lines for large-scale transposon-mutagenesis in tomato
Source: PLoS One. 2025 Nov 19;20(11):e0335612. doi: 10.1371/journal.pone.0335612 (PMC12629433; doi:10.1371/journal.pone.0335612)
Supplement: S8 Fig — (PDF) [file pone.0335612.s008.pdf]

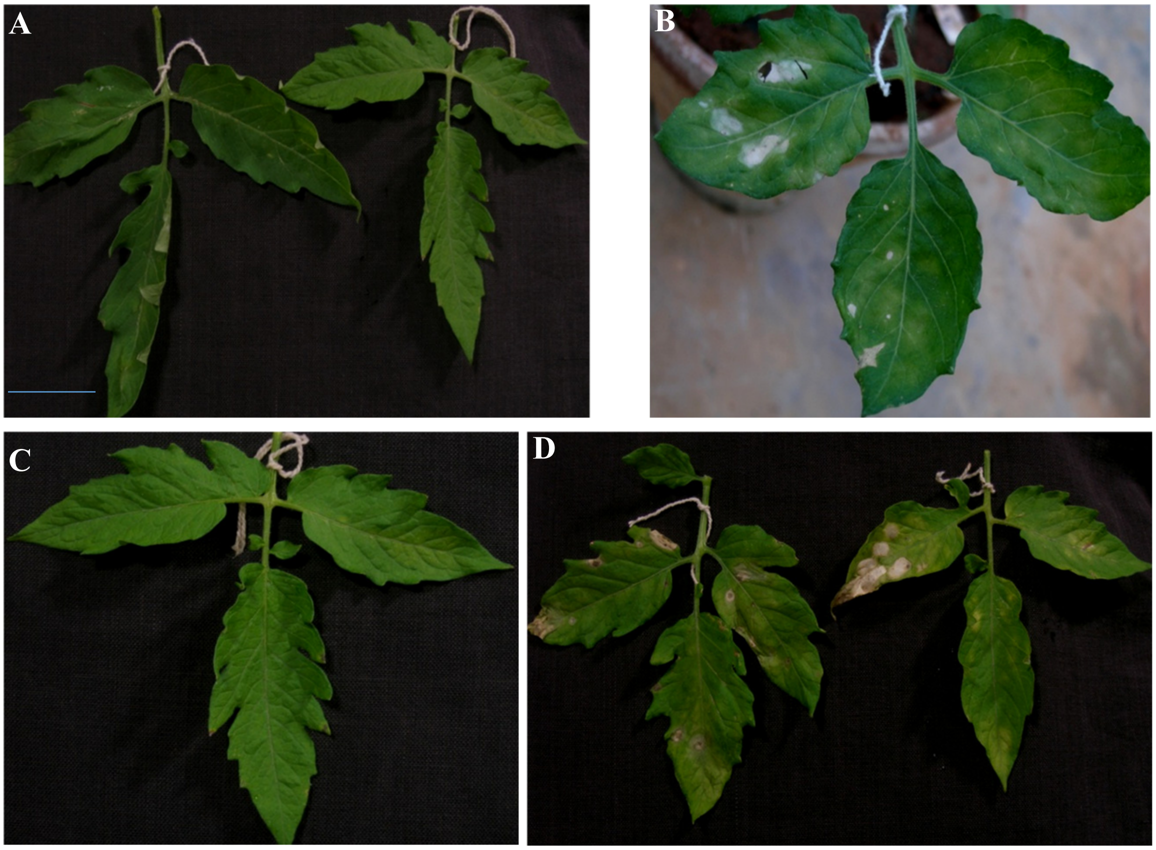

S8 Fig. Kanamycin painting assay for identification of transgenic plants. **A-B.** The wild-type leaf after ten days of mock painting (**A**) and kanamycin painting (**B**). **C.** Leaf of a transgenic line exhibiting resistance to kanamycin. **D.** Leaf of a transgenic line exhibiting sensitivity to kanamycin. Note the appearance of bleached spots at the point of kanamycin application.
