# Supplementary material for: Development of Ac- and Ds-tagged starter lines for large-scale transposon-mutagenesis in tomato
Source: PLoS One. 2025 Nov 19;20(11):e0335612. doi: 10.1371/journal.pone.0335612 (PMC12629433; doi:10.1371/journal.pone.0335612)
Supplement: S9 Fig — (PDF) [file pone.0335612.s009.pdf]

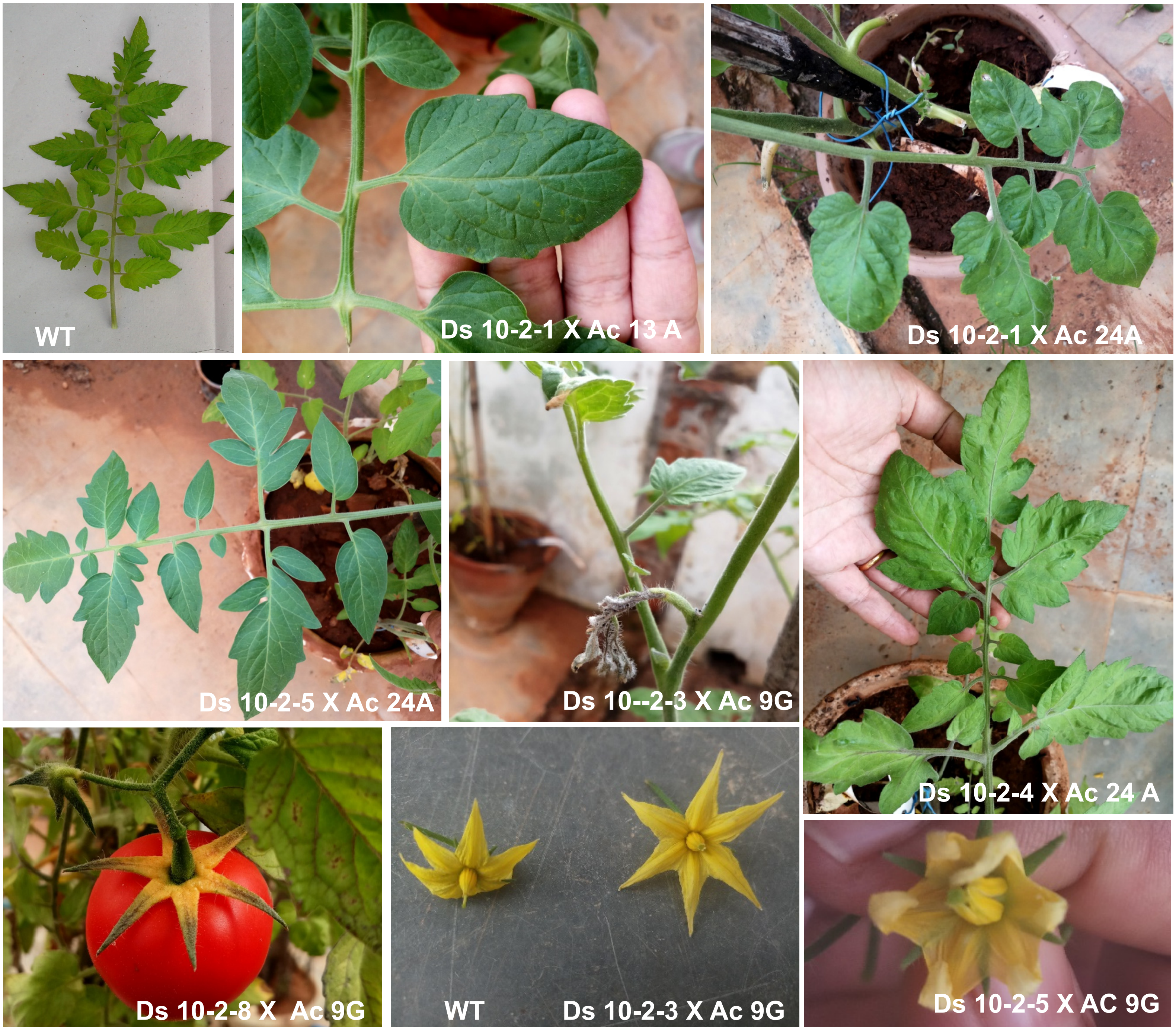

S9 Fig. Phenotypic alterations in F_2 progeny of *Ac* X *Ds* crosses compared to Arka Vikas (WT). The number on the bottom of each picture indicates the name of the cross between *Ac* and *Ds* parents, **WT**, Untransformed plant. Note potato leaf phenotypes, leathery leaf phenotypes, fleshy sepals, introse stamen, and altered anther cone structure.
