# Supplementary figures and images for: Development of Ac- and Ds-tagged starter lines for large-scale transposon-mutagenesis in tomato
Source: PLoS One. 2025 Nov 19;20(11):e0335612. doi: 10.1371/journal.pone.0335612 (PMC12629433; doi:10.1371/journal.pone.0335612)

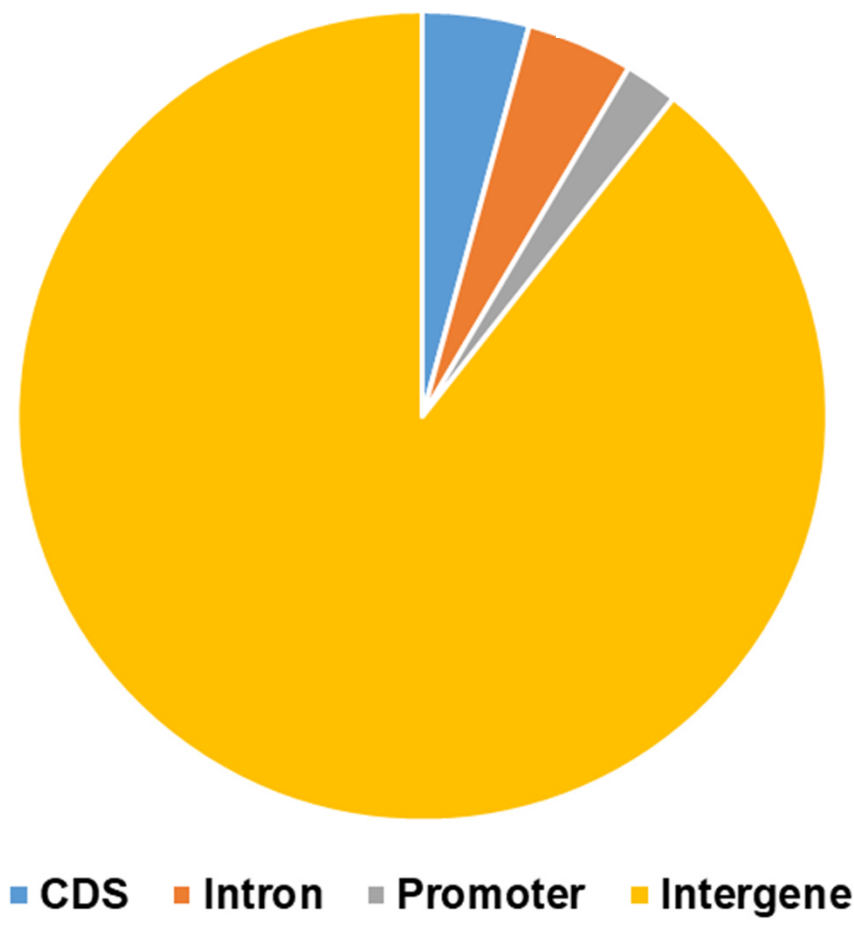

**S10 Fig.** Distribution of *Ds*-insertion sites in Intergenic and Intragenic regions.

Supplement: S10 Fig — (PDF) [file pone.0335612.s010.pdf]
