# Supplementary material for: Development of Ac- and Ds-tagged starter lines for large-scale transposon-mutagenesis in tomato
Source: PLoS One. 2025 Nov 19;20(11):e0335612. doi: 10.1371/journal.pone.0335612 (PMC12629433; doi:10.1371/journal.pone.0335612)
Supplement: S1 Table — (PDF) [file pone.0335612.s011.pdf]

S1 Table: Composition of MS media used in various transformations and regeneration steps.

**Components**	**Seed germination**	**Pre-culture and co-cultivation**	**Selection**	**Rooting**
MS Salts	0.5 X	1 X	1 X	1 X
B5 Vitamins	0.5 X	1 X	1 X	1 X
Sucrose (g/L)	15	30	30	30
Agar (% w/v)	0.8	0.8	0.8	0.8
BAP mg/L	0	2	0	0
IAA mg/L	0	0	0.1	0
Zeatin mg/L	0	0	1	0
Kanamycin mg/L	0	0	100	100
Cefotaxime mg/L	0	0	500	500
