# Supplementary material for: Development of Ac- and Ds-tagged starter lines for large-scale transposon-mutagenesis in tomato
Source: PLoS One. 2025 Nov 19;20(11):e0335612. doi: 10.1371/journal.pone.0335612 (PMC12629433; doi:10.1371/journal.pone.0335612)
Supplement: S2 Table — (PDF) [file pone.0335612.s012.pdf]

S2 Table : Step-wise survival of explants used for tomato transformation.

A. From ~700 explants,at the end we obtained 24 independent *Ac-TPase* transgenic lines

**Stage**	**No. of explants**
Explants used at pre-culture stage	~700
Explants surviving at callusing stage	~430
Explants surviving at selection stage	~40
Explants able to set root	24
Putative transgenic lines transferred to green house	24
Transformation frequency	~3.1 %

B. From ~830 explants, at the end we obtained 20 independent Ds transgenic line

**Stage**	**No. of explants**
Explants used at pre-culture stage	~830
Explants surviving at callusing stage	~450
Explants surviving at selection stage	~55
Explants able to set root	22
Putative transgenic lines transferred to green house	20
Transformation frequency	~2.5%
