# Supplementary material for: Development of Ac- and Ds-tagged starter lines for large-scale transposon-mutagenesis in tomato
Source: PLoS One. 2025 Nov 19;20(11):e0335612. doi: 10.1371/journal.pone.0335612 (PMC12629433; doi:10.1371/journal.pone.0335612)
Supplement: S3 Table — (PDF) [file pone.0335612.s013.pdf]

S3 Table : The sequences of primers were for PCR. The PCR product size of the respective primer pair and cycling conditions are also mentioned next to the respective primer pairs.

Gene	Primer orientation	Sequence (5′→3′)	Product size	PCR Conditions
Ac-TPase element	FP	CTCACTGAACTCCTATCT	586 bp	At 94 °C =4 min, 94 °C =30 sec, 53 °C= 50 sec, 72 °C =1 min, 72 °C= 8 min, 12 °C = ∞, 30 cycles
	RP	ACTCAGAAGCAACAGTTGAC		
sGFP For clonning	FP	AATTATCTAGAATGGTGAGCAAGGGCGAGGAGCTG	700 bp	Primer Tm 58°C= 50 sec,
	RP	CCTAAGGTACCTACTTGTACAGCTCGTCCATGCCGT		
NPTII	FP	CGGTCATTTCGAACCCCAGA	750 bp	Primer Tm 56°C= 50 sec,
	RP	GATGGATTGCACGCAGGTTC		
NPTII NOSter	FP	CGGTCATTTCGAACCCCAGA	~1 kb	Primer Tm 55°C= 1 min
	RP	GCCGGTCTTGCGATGATTATCAT		
AtUBI Promoter	FP	AATTAGGATCCAACTCAACACACAGCCTGATCC	2012 bp	Primer Tm 54°C= 2 min
	RP	CCTAATCTAGAAACAAAAATTGGAGATCAGATAC		
RFP	FP	GACGTCATCAAGGAGTTCAT	~ 500 bp	Primer Tm 51°C= 50 sec
	RP	ATAGTCTTCTTCTGCATTACGG		
CaMV35S promoter	FP	AGATTAGCCTTTTCAATTTCAG	841 bp	Primer Tm 51°C= 1 min
	RP	AGTCCCCCGTGTTCTCTCCA		
ChvA	FP	CGAAACGCTGTTCGGCCTGTGG	876 bp	Primer Tm 56°C= 1 min
	RP	GTTCAGCAGGCCGGCATCCTGG		
CYCB	FP	GGGCTCAATTCGACGTGATC	675 bp	Primer Tm 55°C= 50 sec
	RP	ACAGGACGACTCACCAAAGA		
NPTII	FP	TGCTCGACGTTGTCACTGAAGC	339 bp	Primer Tm 51°C= 30 sec
	RP	AGCAGGCATCGCCATGGGTCAC		
sGFP (for screening)	FP	GCTGACCCTGAAGTTCATCT	331bp	Primer Tm 51°C= 30 sec
	RP	ATAGACGTTGTGGCTGTTGTAG		
