# Supplementary material for: Development of Ac- and Ds-tagged starter lines for large-scale transposon-mutagenesis in tomato
Source: PLoS One. 2025 Nov 19;20(11):e0335612. doi: 10.1371/journal.pone.0335612 (PMC12629433; doi:10.1371/journal.pone.0335612)
Supplement: S4 Table — (PDF) [file pone.0335612.s014.pdf]

S4 Table: List and sequences of primers were used in FPNI-PCR. The PCR step at which the primers were used is mentioned at the right side of the respective primer.

Name of Primer	Primer sequence (5′---3′)	PCR in which primers has been used
FP 1:	GTAATACGACTCACTATAGGGCACGCGTGGTNTCGASTWTS GWGTT	1st PCR primer
FP 2:	GTAATACGACTCACTATAGGGCACGCGTGGTNGTCGASWGANAWGAA	1st PCR primer
FP 3:	GTAATACGACTCACTATAGGGCACGCGTGGTWGTGNAGWANCANAGA	1st PCR primer
FP 4:	GTAATACGACTCACTATAGGGCACGCGTGGTAGWGNAGWANCAWAGG	1st PCR primer
FP 5:	GTAATACGACTCACTATAGGGCACGCGTGGTNGTAWAASGTNTSCAA	1st PCR primer
FP 6:	GTAATACGACTCACTATAGGGCACGCGTGGTNGACGASWGANAWGAC	1st PCR primer
FP 7:	GTAATACGACTCACTATAGGGCACGCGTGGTNGACGASWGANAWGAA	1st PCR primer
FP 8:	GTAATACGACTCACTATAGGGCACGCGTGGTGTNCGASWCANAWGTT	1st PCR primer
FP 9:	GTAATACGACTCACTATAGGGCACGCGTGGTNCAGCTWSCTNTSCTT	1st PCR primer
FSP1:	GTAATACGACTCACTATAGGGC	2nd PCR primer
FSP2:	ACTATAGGGCACGCGTGGT	3rd PCR primer
PLB1	AAATCACCACTCGATACAGGCA	1st PCR primer
PLB2	ACCACAATATATCCTGCCACCA	2nd PCR primer
PLB3	GCGTCAATTTGTTTACACCA	3rd PCR primer
PRB1	TTGACAGGATATATTGGCGGGT	1st PCR primer
PRB2	AAGGGCGTGAAAAGGTTTATCC	2nd PCR primer
PRB3	CCATTTGTATGTGCATGCCAAC	3rd PCR primer
