# Supplementary material for: Development of Ac- and Ds-tagged starter lines for large-scale transposon-mutagenesis in tomato
Source: PLoS One. 2025 Nov 19;20(11):e0335612. doi: 10.1371/journal.pone.0335612 (PMC12629433; doi:10.1371/journal.pone.0335612)
Supplement: S5 Table — (PDF) [file pone.0335612.s015.pdf]

S5 Table, Sequences of primers used for inverse PCR. The step at which the primers were used is mentioned at the right side of the respective primer.

**Name of primer**	**Primer Sequence (5′→ 3′)**	**Step where primer**
Ds 5-1	CCGTTTACCGTTTTGTATATCCCG	1st PCR primer
Ds 5-2	CGTTCCGTTTTCGTTTTTTACC	2nd PCR primer
Ds 5-3	CGGTCGGTACGGGATTTTCC	3rd PCR primer
Ds 3-1	CGATTACCGTATTTATCCCGTTCG	1st PCR primer
Ds 3-2	CCGGTATATCCCGTTTTCG	2nd PCR primer
Ds 3-3	GAAAATGAAAACGGTAGAGGT	3rd PCR primer
