# Supplementary material for: Development of Ac- and Ds-tagged starter lines for large-scale transposon-mutagenesis in tomato
Source: PLoS One. 2025 Nov 19;20(11):e0335612. doi: 10.1371/journal.pone.0335612 (PMC12629433; doi:10.1371/journal.pone.0335612)
Supplement: S6 Table — (PDF) [file pone.0335612.s016.pdf]

S6 Table: Kanamycin resistance assay of *Ac-TPase* starter lines

Line Numbers	Sensitive	Resistant	PCR Positive lines	Southern Positive lines
Ac-TPase - 1A	*Ac-TPase* -1A1, 1A2, 1A3, 1A4, 1A7	*Ac-TPase* - 1A5,1A6	*Ac-TPase* - 1A5, 1A6	***Ac-TPase* - 1A6**
Ac-TPase - 2C	*Ac-TPase* - 2C3	*Ac-TPase* - 2C1, 2C2, 2C4, 2C5, 2C6, 2C7, 2C8, 2C9, 2C10	*Ac-TPase* - 2C1, 2C2, 2C6, 2C8, 2C9, 2C10	***Ac-TPase* - 2C1, 2C2**
Ac-TPase - 3A	*Ac-TPase* - 3A1, 3A4	*Ac-TPase* - 3A2, 3A3, 3A5, 3A6	*Ac-TPase* - 3A2,3A3,3A6	
Ac-TPase - 7B	*Ac-TPase* -7B1, 7B5, 7B6	*Ac-TPase* - 7B3, 7B7, 7B9	*Ac-TPase* - 7B3, 7B7,7B9	
Ac-TPase - 9G	*Ac-TPase* - 9G3, 9G5, 9G6, 9G8	*Ac-TPase* - 9G1, 9G2, 9G4, 9G7, 9G9, 9G10	*Ac-TPase* - 9G1, 9G2, 9G9,9G10	***Ac-TPase* 9G9**
Ac-TPase - 13A	*Ac-TPase* - 13A7	*Ac-TPase* - 13A1, 13A2, 13A3, 13A4, 13A5, 13A6	*Ac-TPase* - 13A1, 13A2, 13A3, 13A5	
Ac-TPase - 13D	*Ac-TPase* - 13D3, 13D4	*Ac-TPase* - 13D1, 13D2, 13D5, 13D6, 13D7, 13D8	*Ac-TPase* - 13D1, 13D2, 13D5, 13D7	
Ac-TPase - 21A	*Ac-TPase* - 21A1, 21A2, 21A4	*Ac-TPase* - 21A3, 21A5, 21A6	*Ac-TPase* - 21A3, 21A5, 21A6	
Ac-TPase - 23D	*Ac-TPase* - 23D3	*Ac-TPase* - 23D1, 23D2, 23D4, 23D6, 23D7, 23D9	*Ac-TPase* - 23D1, 23D2, 23D6, 23D7, 23D9	***Ac-TPase* - 23D2, 23D6**
Ac-TPase - 24A	*Ac-TPase* - 24A1, 24A2	*Ac-TPase* - 24A3,24A4	*Ac-TPase* - 24A3, 24A4	***Ac-TPase* - 24A3**
