# Supplementary material for: Development of Ac- and Ds-tagged starter lines for large-scale transposon-mutagenesis in tomato
Source: PLoS One. 2025 Nov 19;20(11):e0335612. doi: 10.1371/journal.pone.0335612 (PMC12629433; doi:10.1371/journal.pone.0335612)
Supplement: S7 Table — (PDF) [file pone.0335612.s017.pdf]

**S7 Table:** The chromosomal location of *Ds* launching pad.

| **S.No.** | **Name of Dis launch pad** | **Chromosome number** | **Location in the genome** | **Sequence ID** |
|---|---|---|---|---|
| 1. | *Ds-2* | 1 | Zinc finger protein VAR3 gene | Solyc01g099230 |
| 2. | *Ds-4* | 9 | Tic 22-like gene | Solyc09g092530 |
| 3. | *Ds-2-2* | 3 | Intergenic | SL3.0ch03, 26676740-26676787 |
