# Supplementary material for: Development of Ac- and Ds-tagged starter lines for large-scale transposon-mutagenesis in tomato
Source: PLoS One. 2025 Nov 19;20(11):e0335612. doi: 10.1371/journal.pone.0335612 (PMC12629433; doi:10.1371/journal.pone.0335612)
Supplement: S8 Table — (PDF) [file pone.0335612.s018.pdf]

S8 Table: Step-wise seedlings screened to find out *Ds* transposition (excision and reinsertion) frequency in the study

**Stage**	**No. of seedlings**
Total no. of F_2 seedlings screened for *Ds* transposition	~1000
No. of seedlings survived	~800
No. of seedlings shows the absence of the *GFP* gene in the PCR	~120 (~15%)
No. of seedlings shows the presence of the *RFP* gene in the PCR	60 (~7.5%)
