# Supplementary material for: Development of Ac- and Ds-tagged starter lines for large-scale transposon-mutagenesis in tomato
Source: PLoS One. 2025 Nov 19;20(11):e0335612. doi: 10.1371/journal.pone.0335612 (PMC12629433; doi:10.1371/journal.pone.0335612)
Supplement: S9 Table — (PDF) [file pone.0335612.s019.pdf]

S9 Table. Distribution of Ds-tagged lines on different chromosomes of tomato.

**Chromosome number**	**Number of Ds transpositions**
1	0
2	4
3	21
4	2
5	1
6	2
7	4
8	9
9	3
10	7
11	2
12	1
