# Supplementary material for: Development of Ac- and Ds-tagged starter lines for large-scale transposon-mutagenesis in tomato
Source: PLoS One. 2025 Nov 19;20(11):e0335612. doi: 10.1371/journal.pone.0335612 (PMC12629433; doi:10.1371/journal.pone.0335612)

S1_Raw images. Original Images of gels/blots used in this study

| No. | Type of Figure | Information | |
|---|---|---|---|
| 1 | Figure 1 AB | Schematic Representation | |
| 2 | Figure 2 ABCDE | Micrographs, Fluorescence imaging station pictures | |
| 3 | Figure 3 | Fluorescence imaging station pictures | |
| 4 | Figure 4 | Gel Image | Provided |
| 5 | Figure 5 | Inverse PCR Gel Images A. B | Provided |
| 6 | Figure 6 | Schematic Representation | |
| 7 | Figure S1 | Flow charts | |
| 8 | Figure S2 | Gel images | Provided |
| 9 | Figure S3 | Schematic Representation | |
| 10 | Figure S4 | A-F Gel Images | Provided |
| 11 | Figure S5 | A-C Gel Images | Unable to locate originals |
| 12 | Figure S6 | Blot pictures | |
| 13 | Figure S7 | Schematic diagram | |
| 14 | Figure S8 | Kanamycin painting assay | |
| 15 | Figure S9 | Plant phenotyping Pictures | |
| 16 | Figure S10 | Pie chart | |

Figure 4A

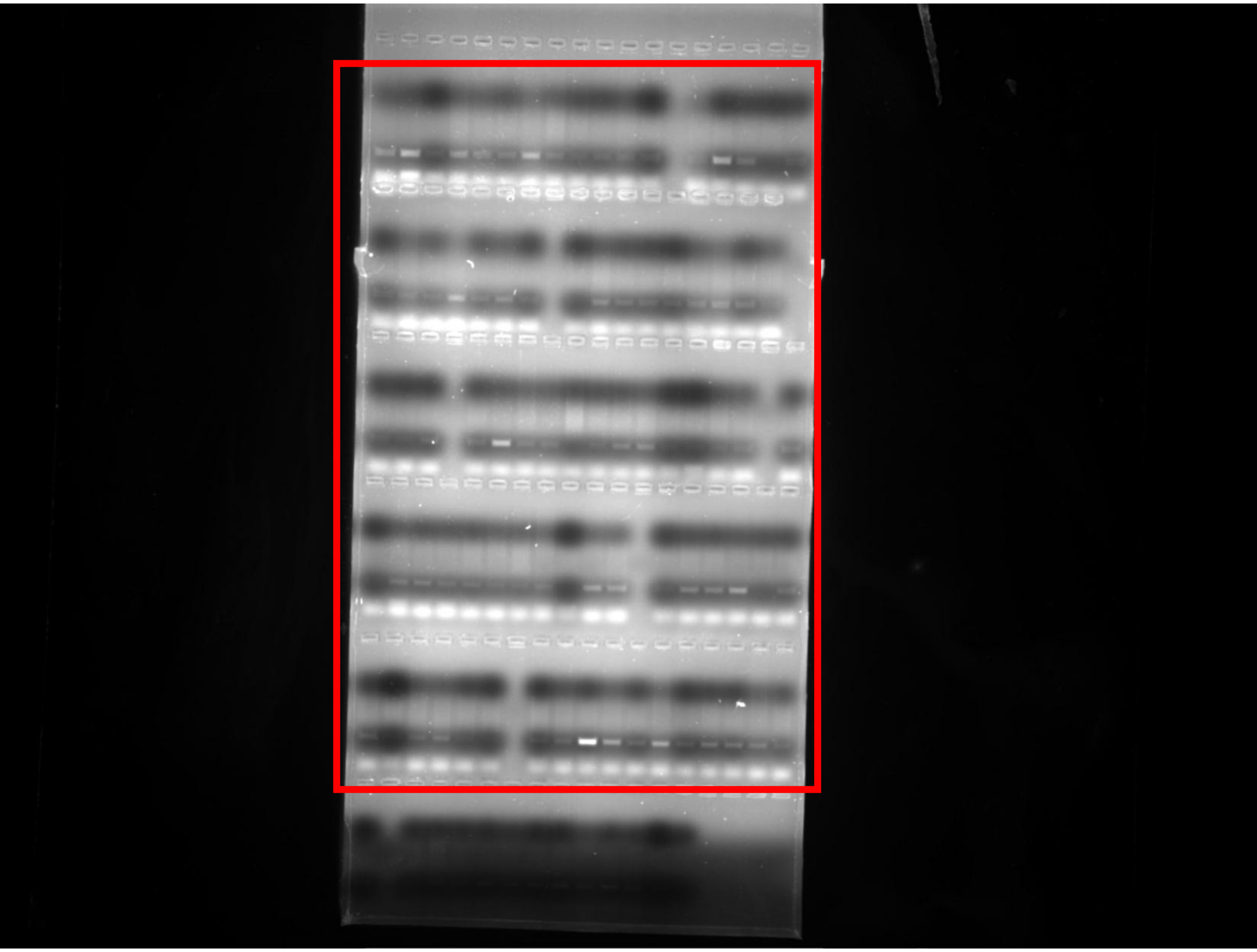

Figure 4B

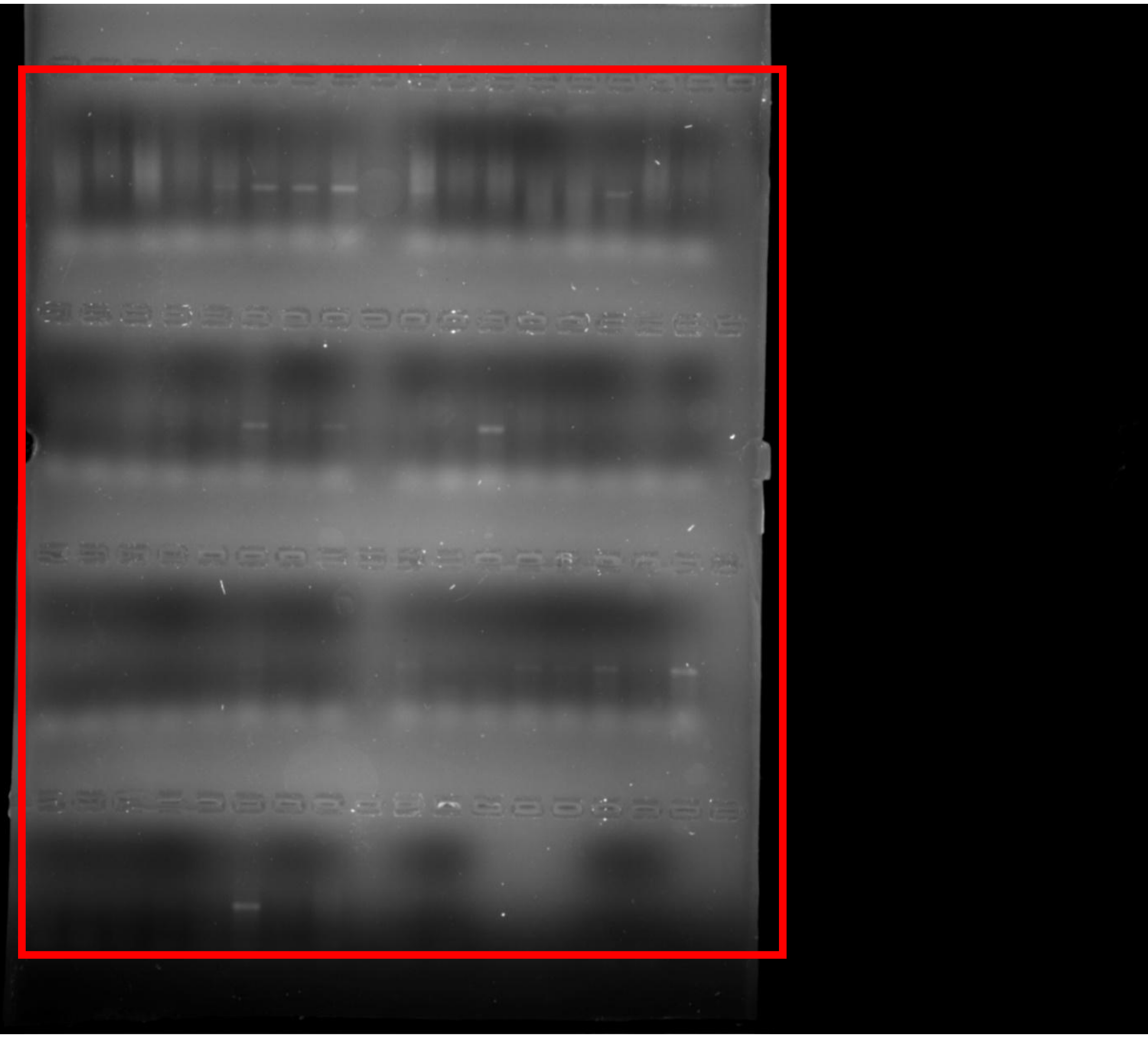

Figure 5A

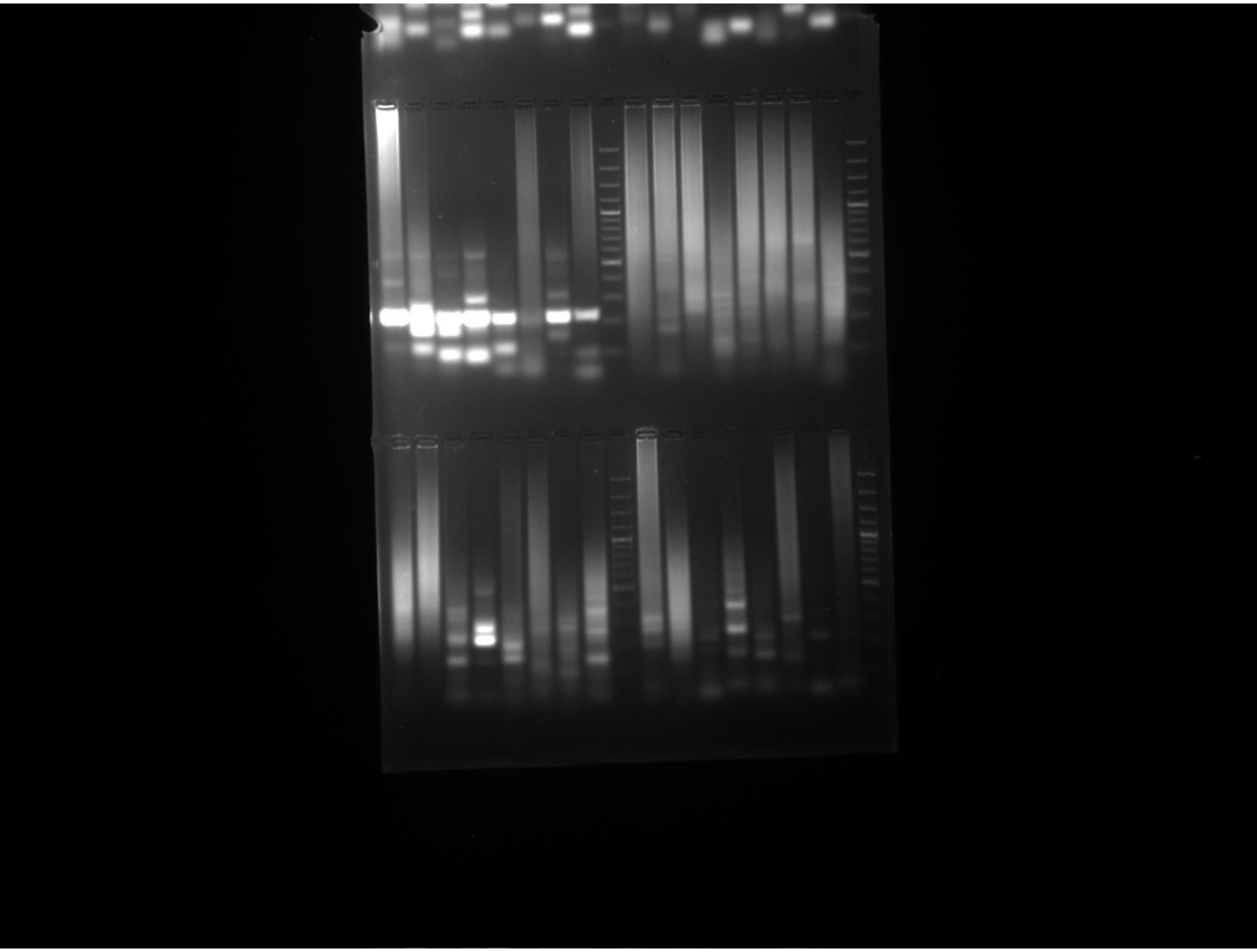

Figure 5B

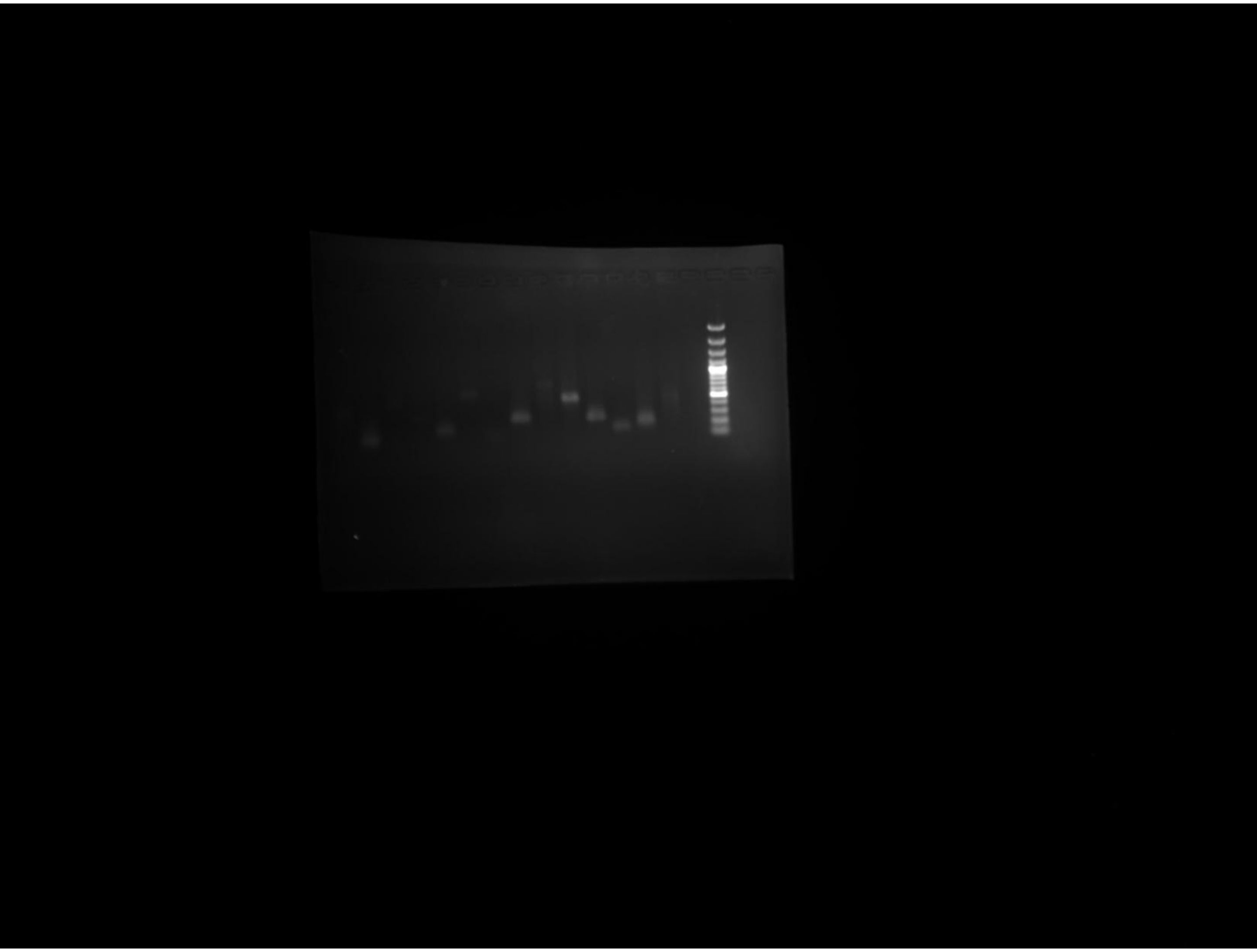

Figure S2A and Figure S2B

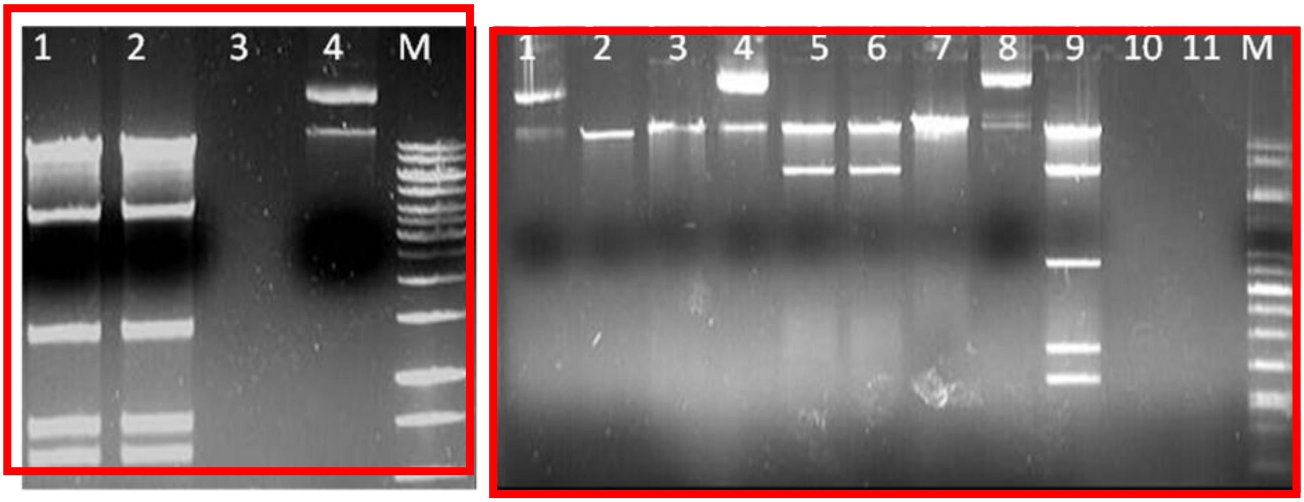

Figure S2C

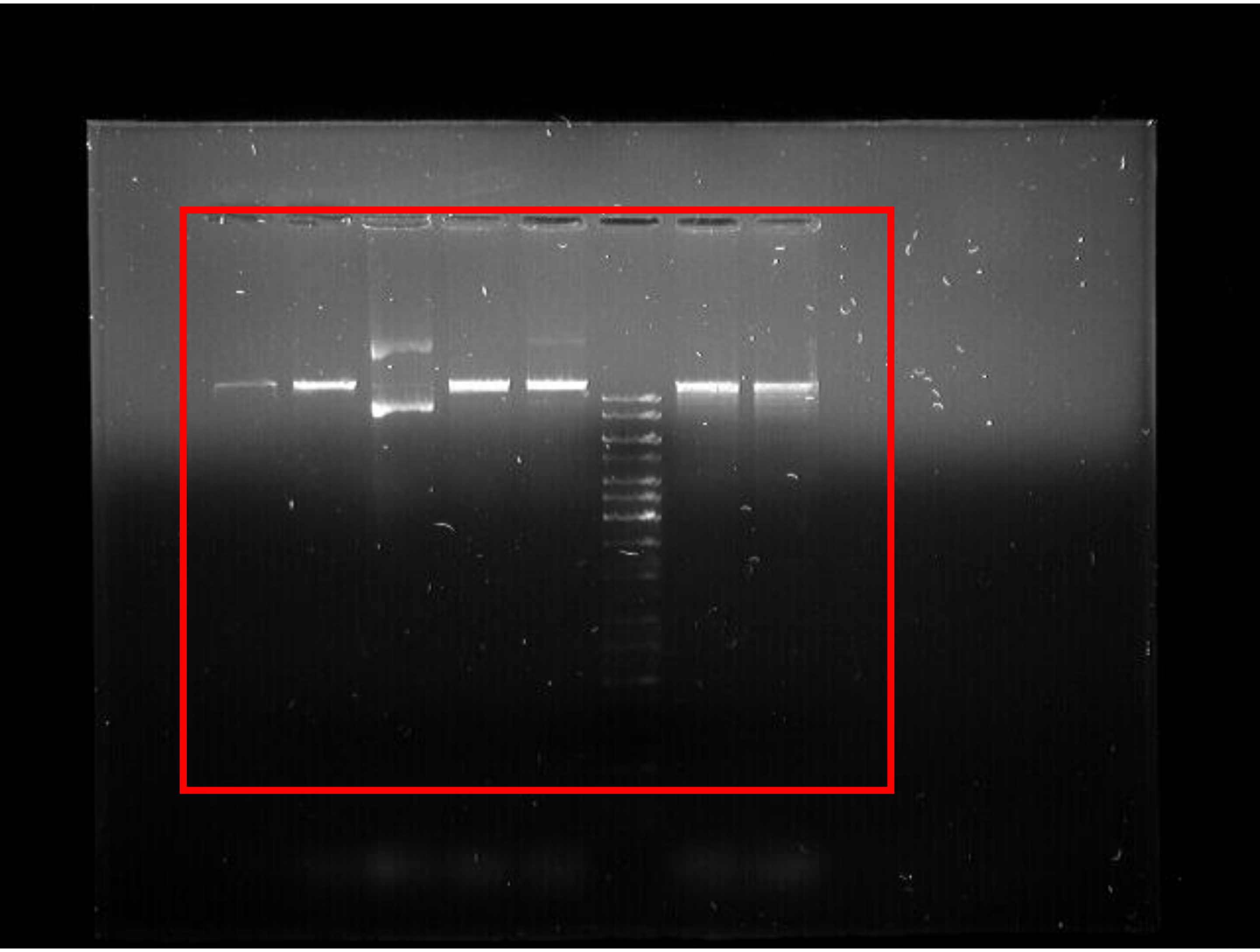

Figure S2D

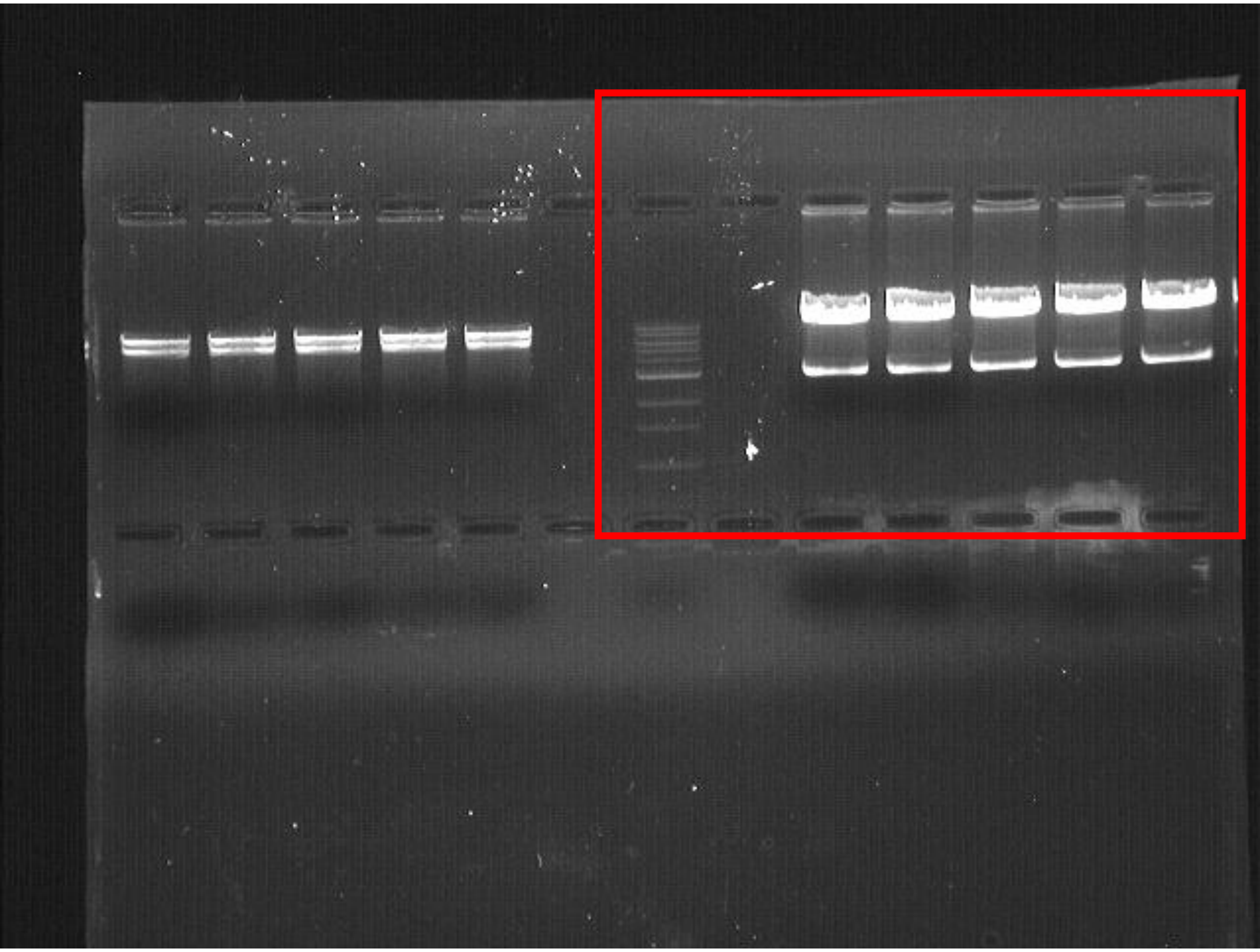

Figure S2E

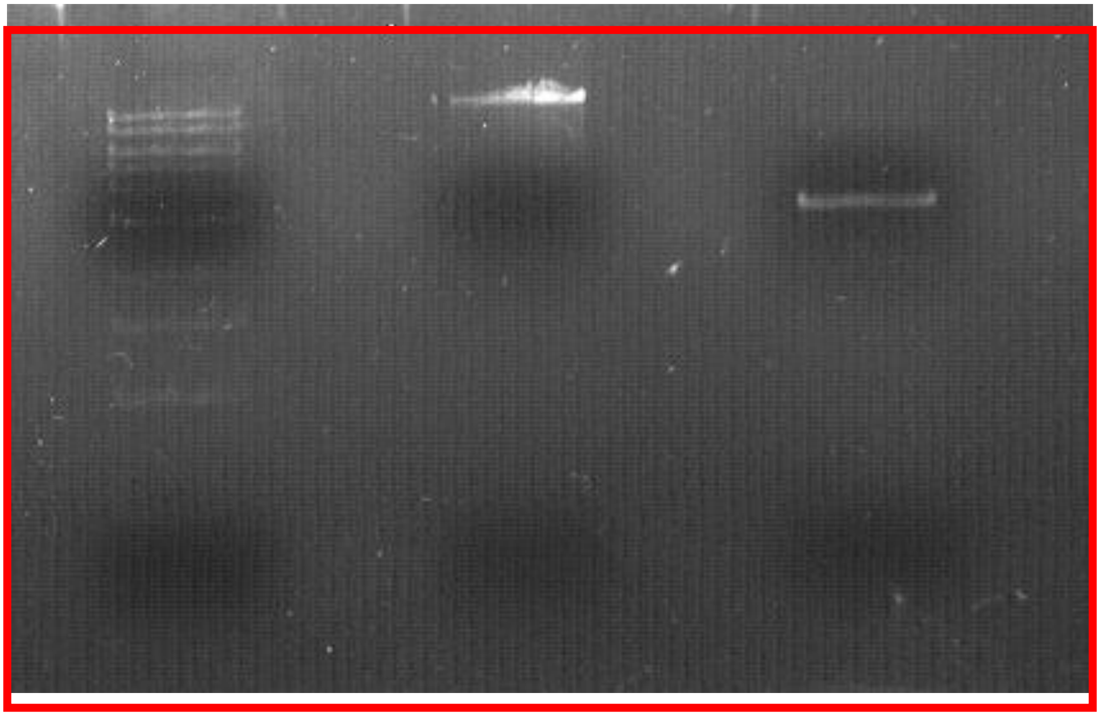

Figure S2G

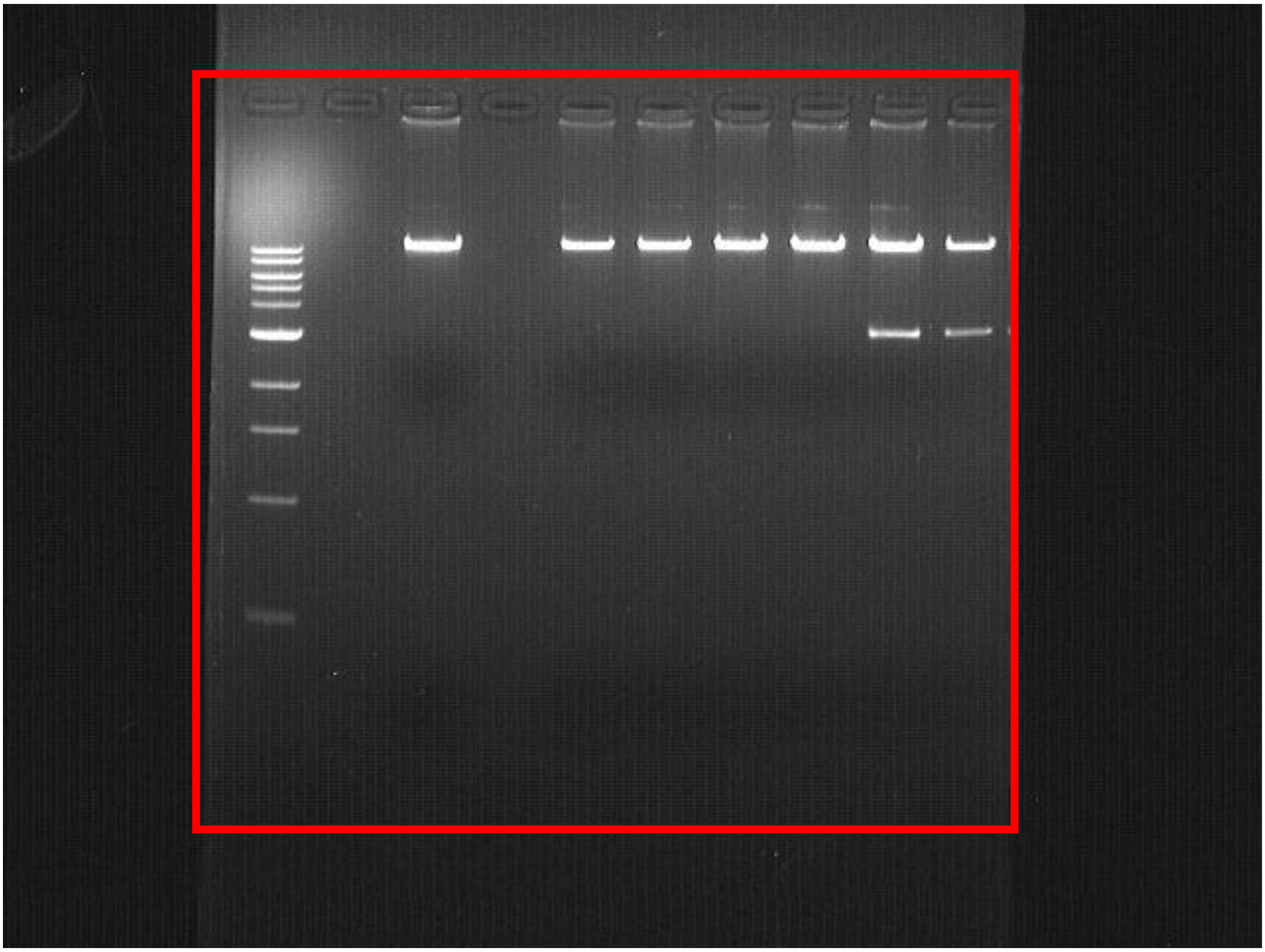

Figure S2H

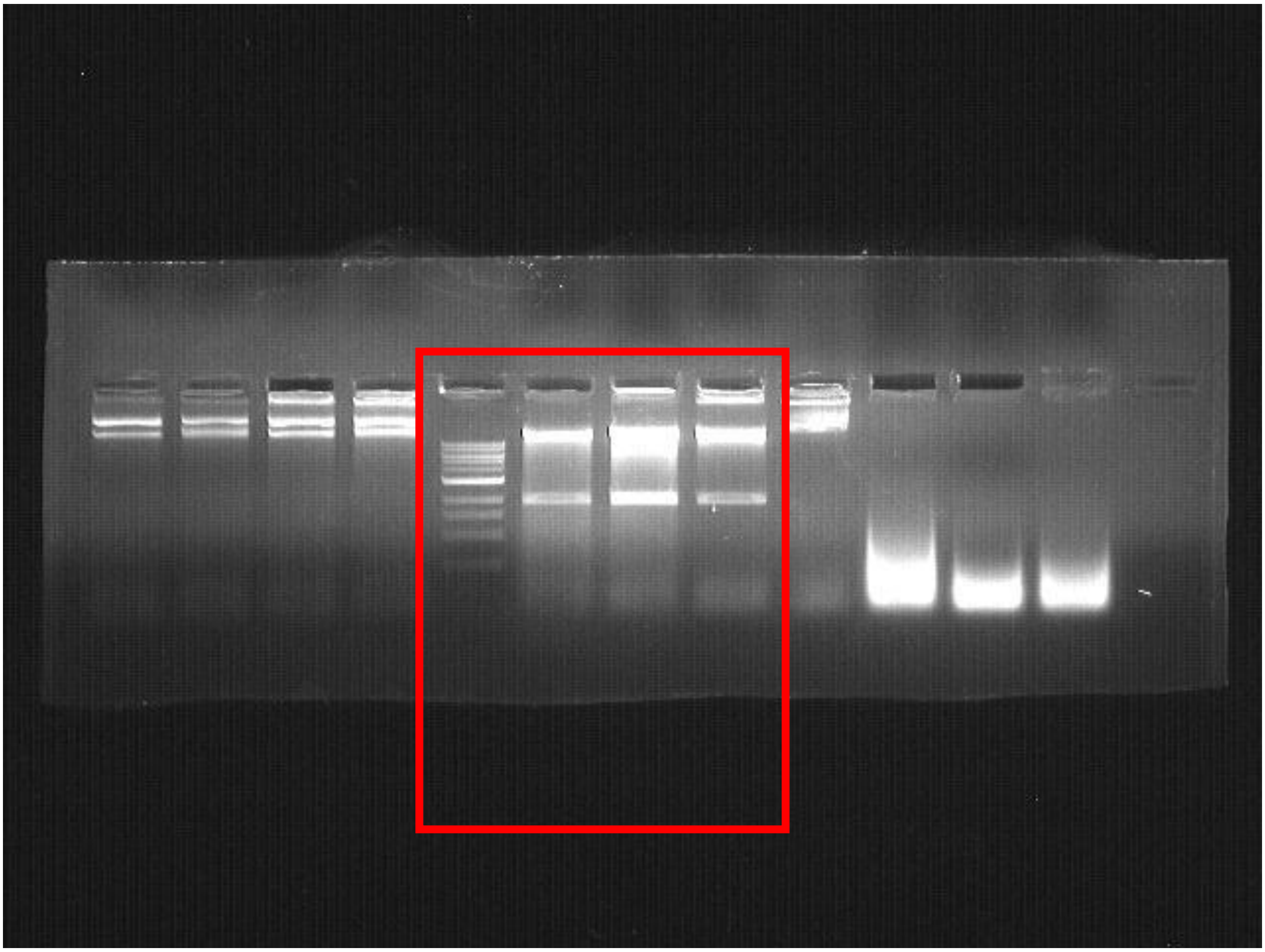

Figure S2J

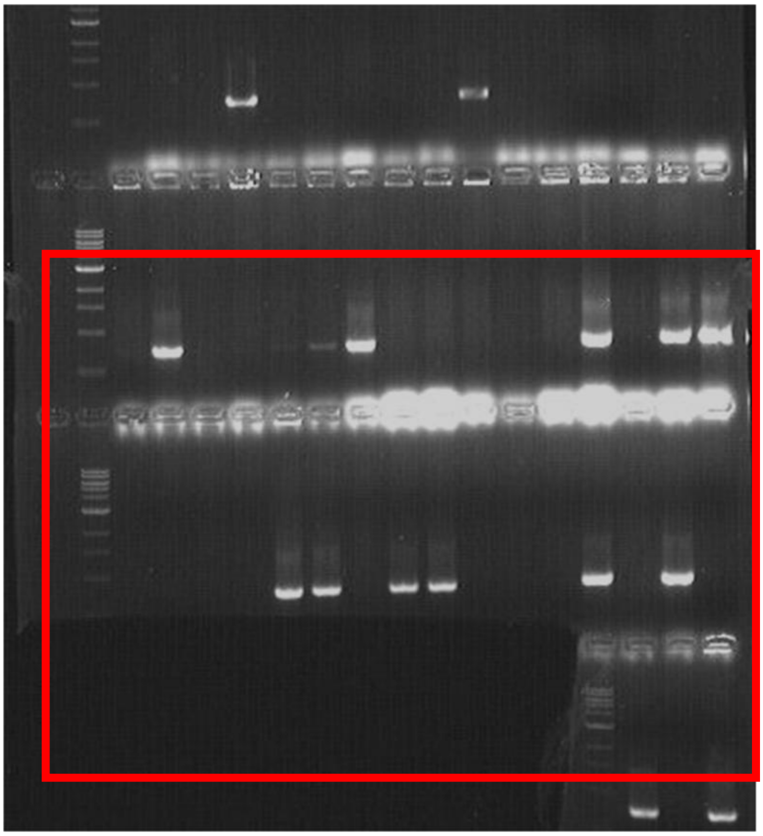

Figure S2K

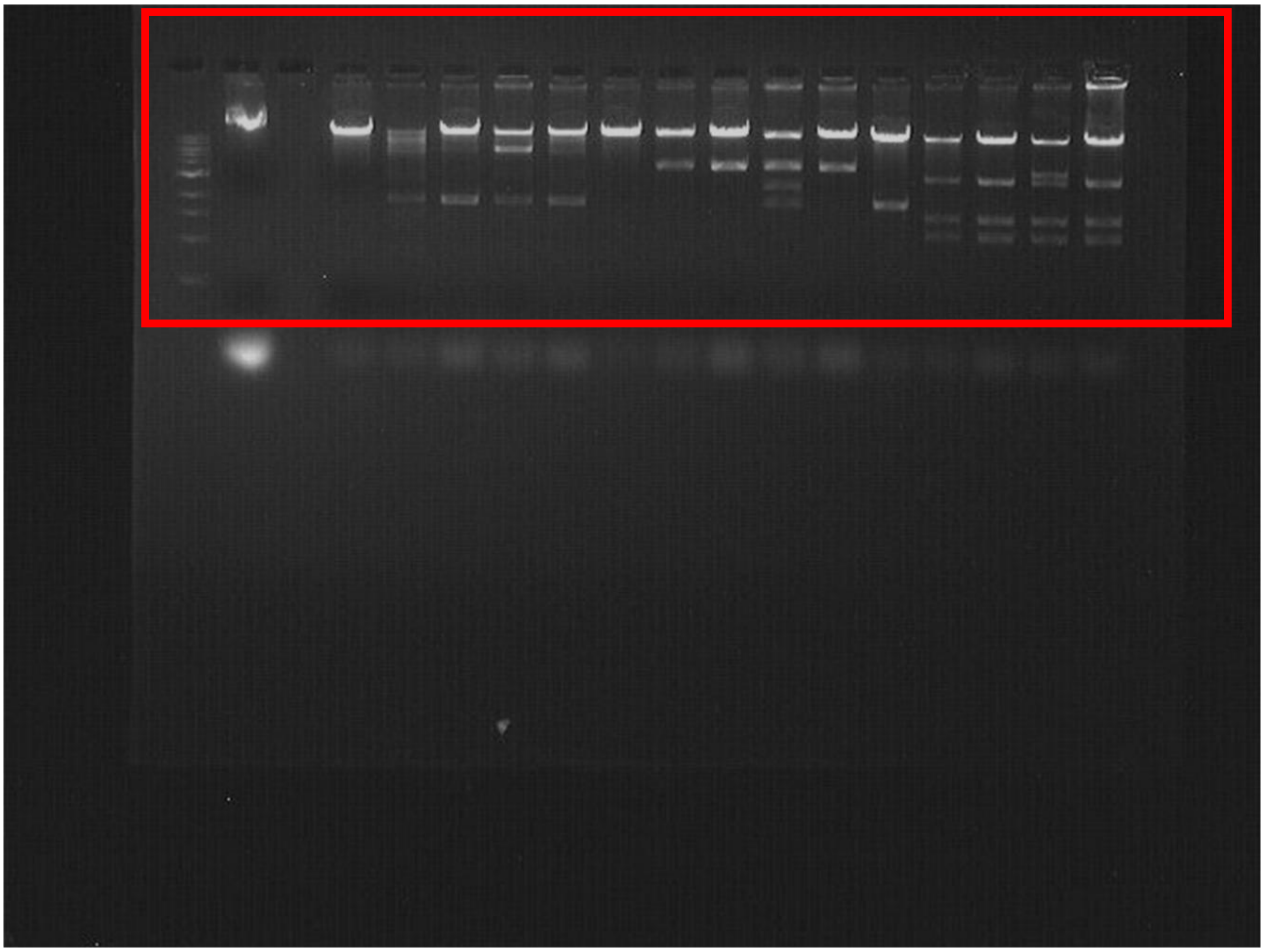

Figure S4A

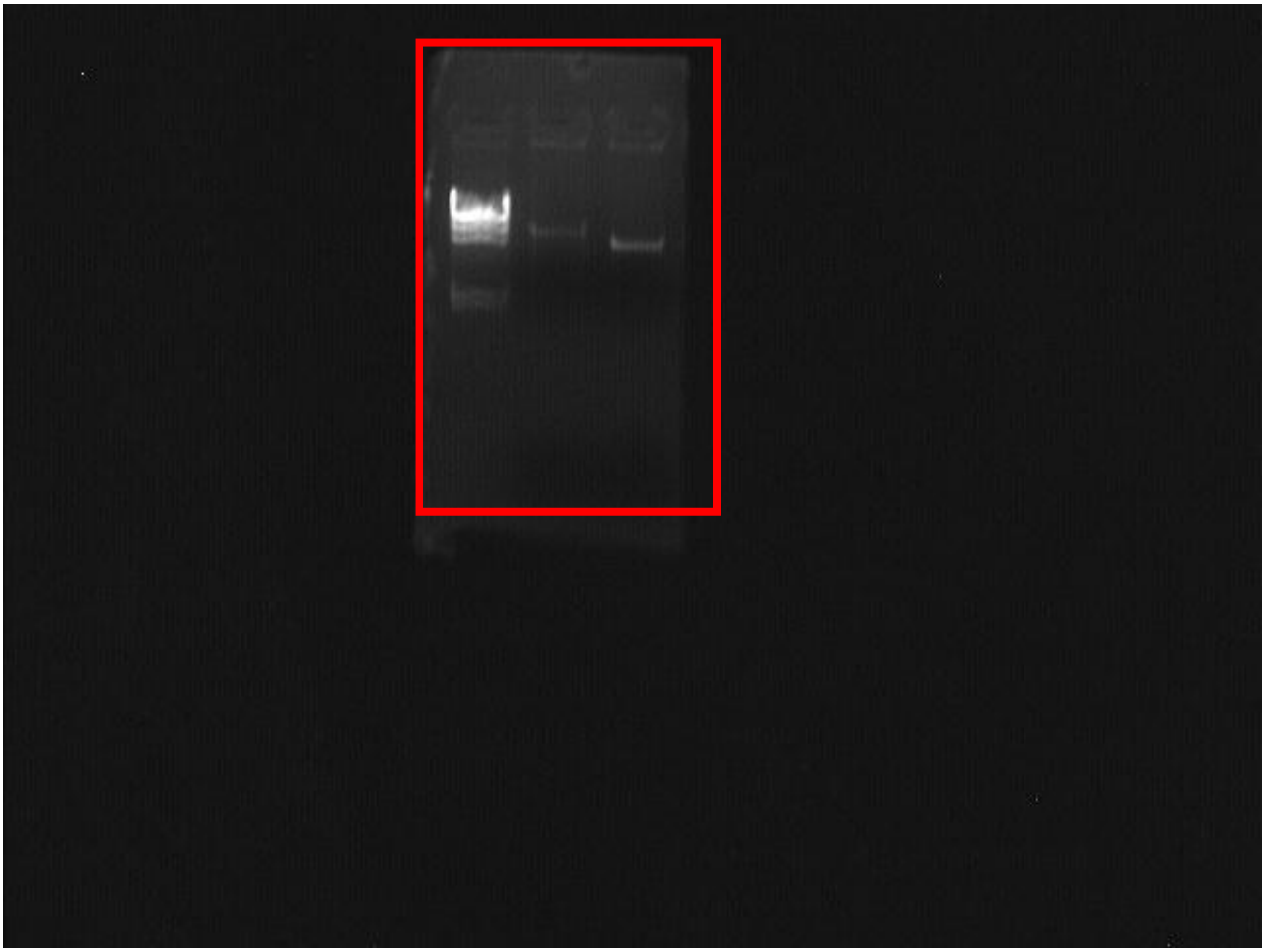

Figure S4B

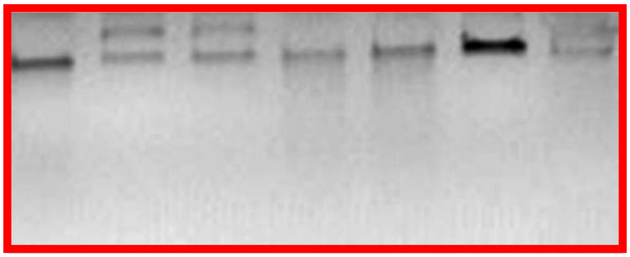

Figure S4C

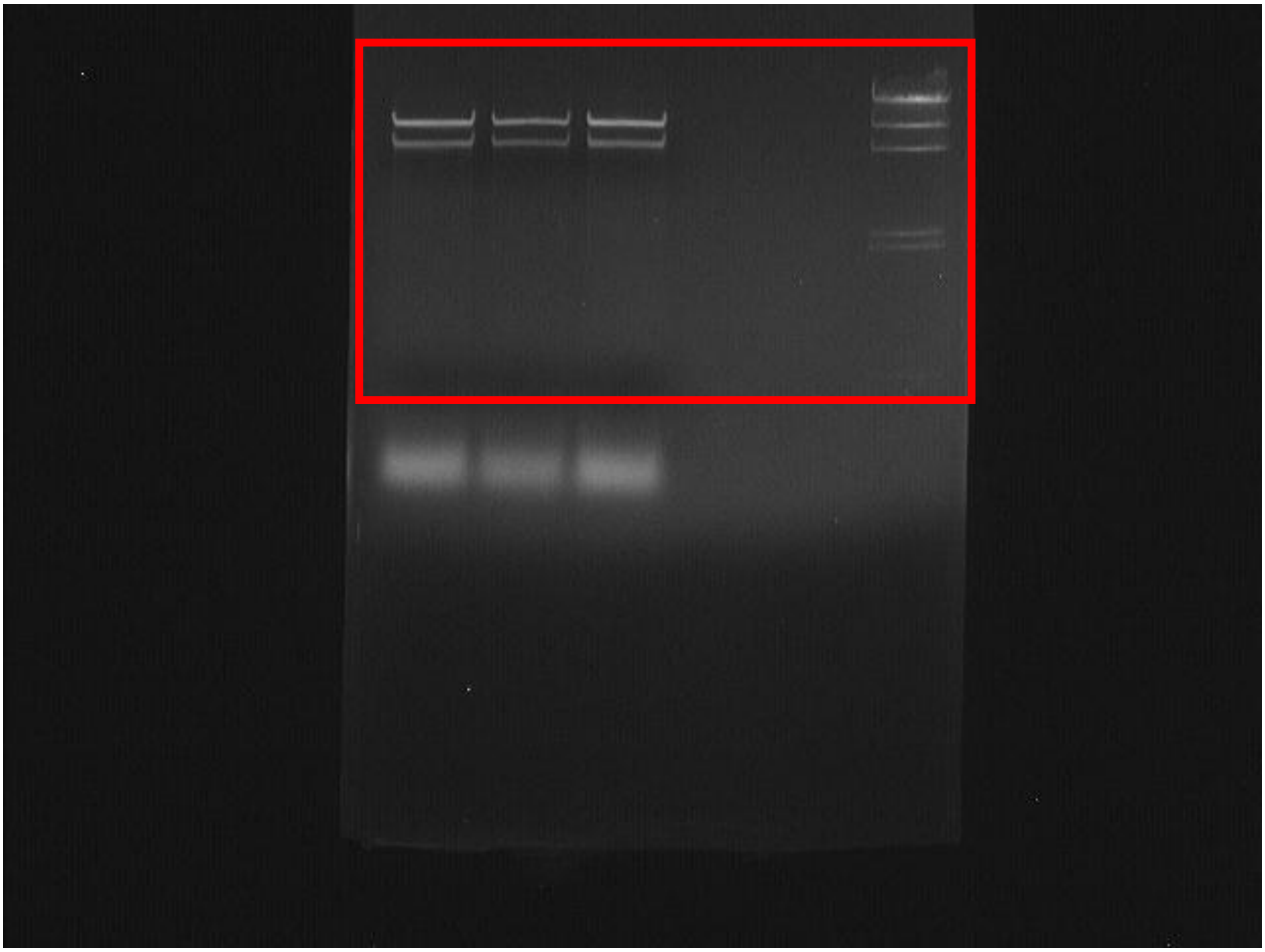

Figure S4D&E

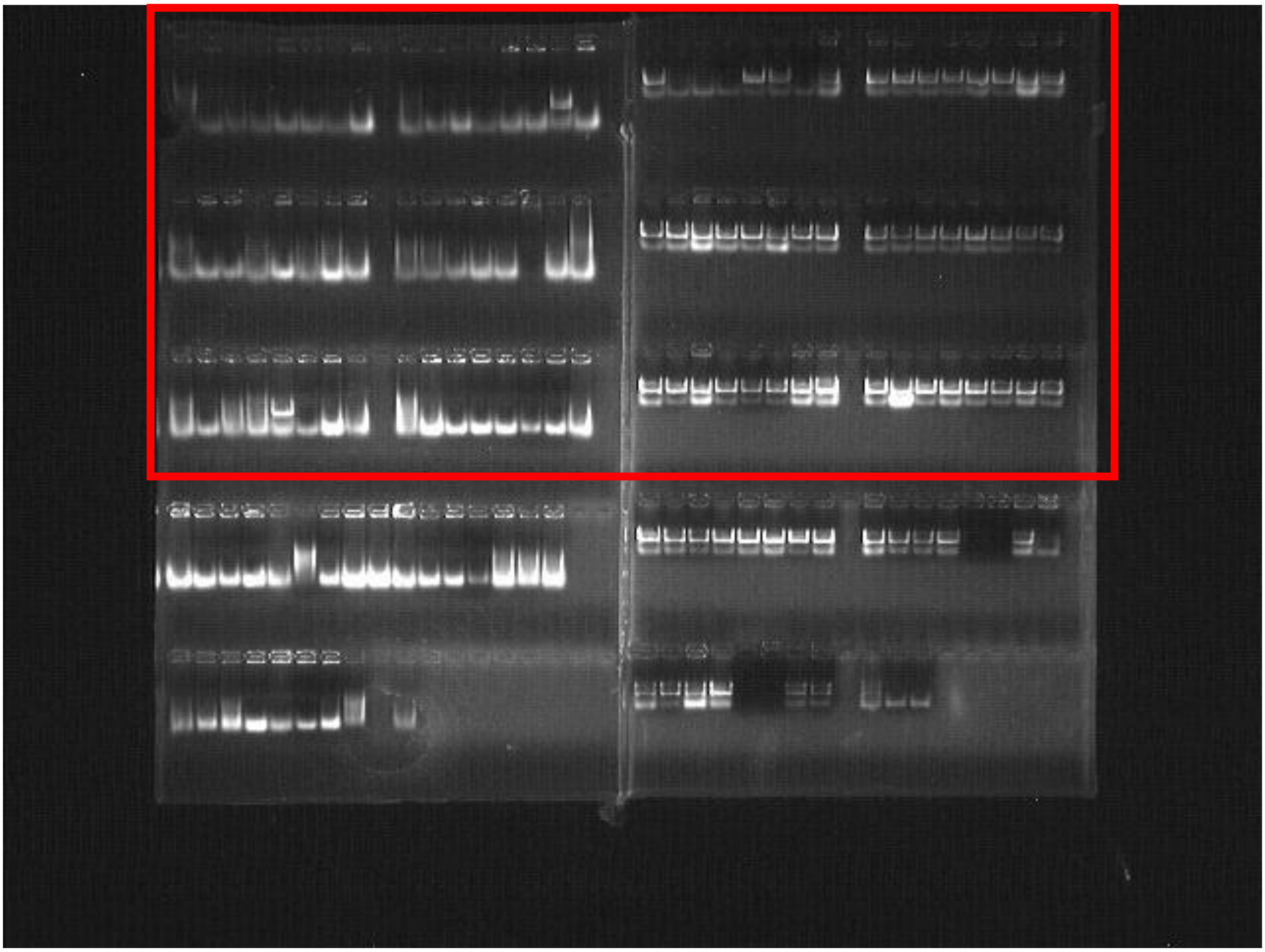

Figure S4F

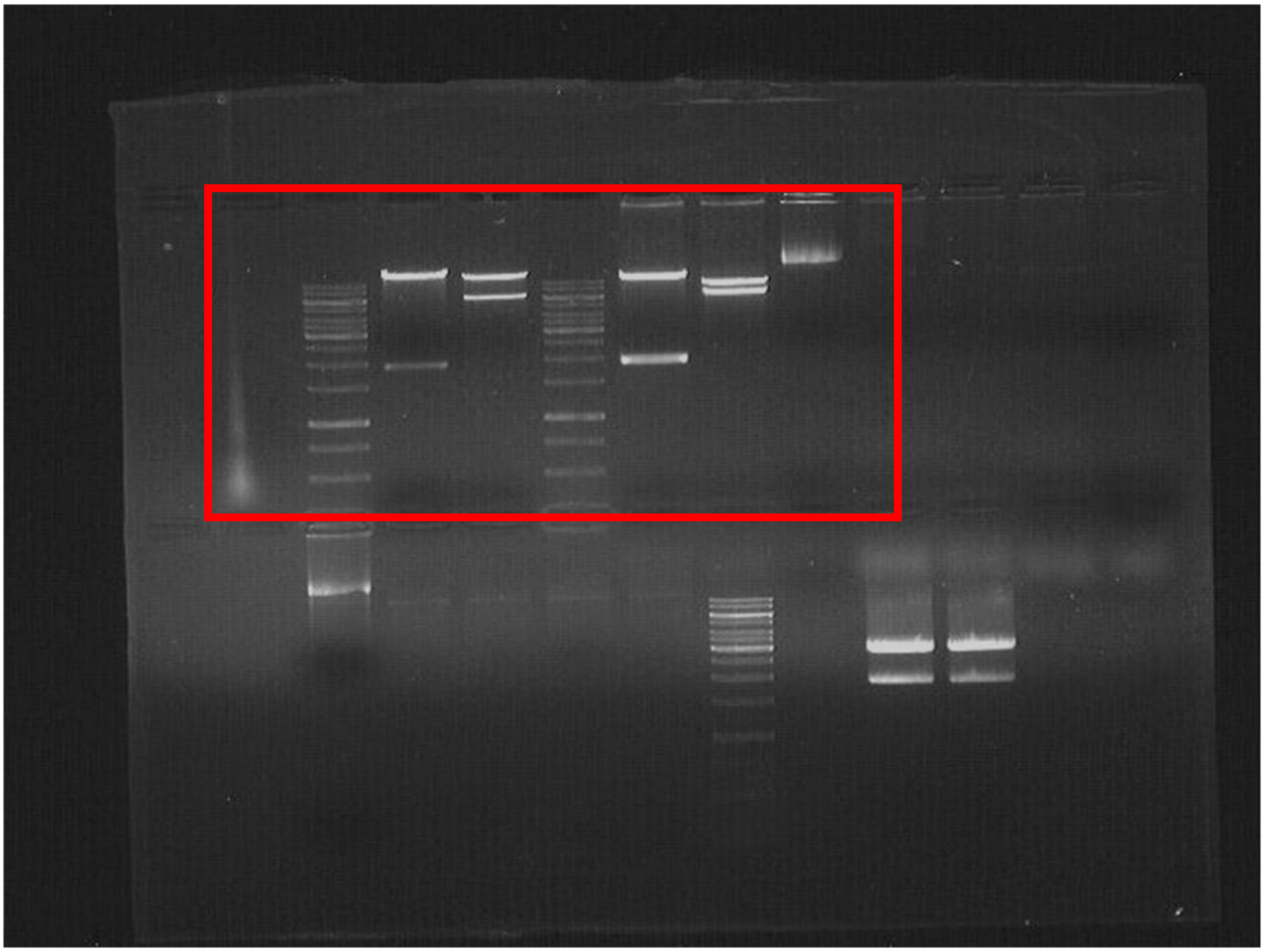

Figure S6A1

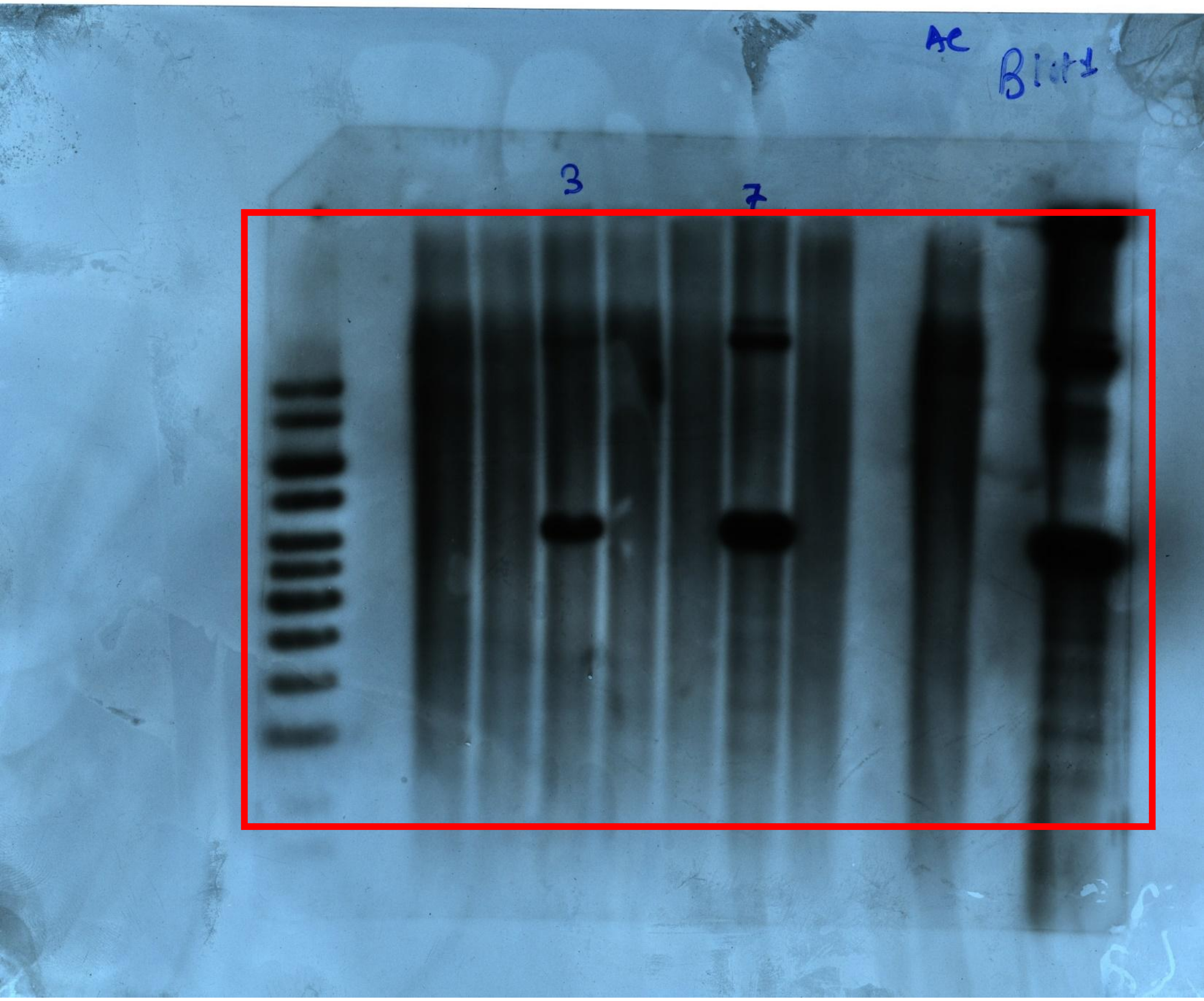

Figure S6A2

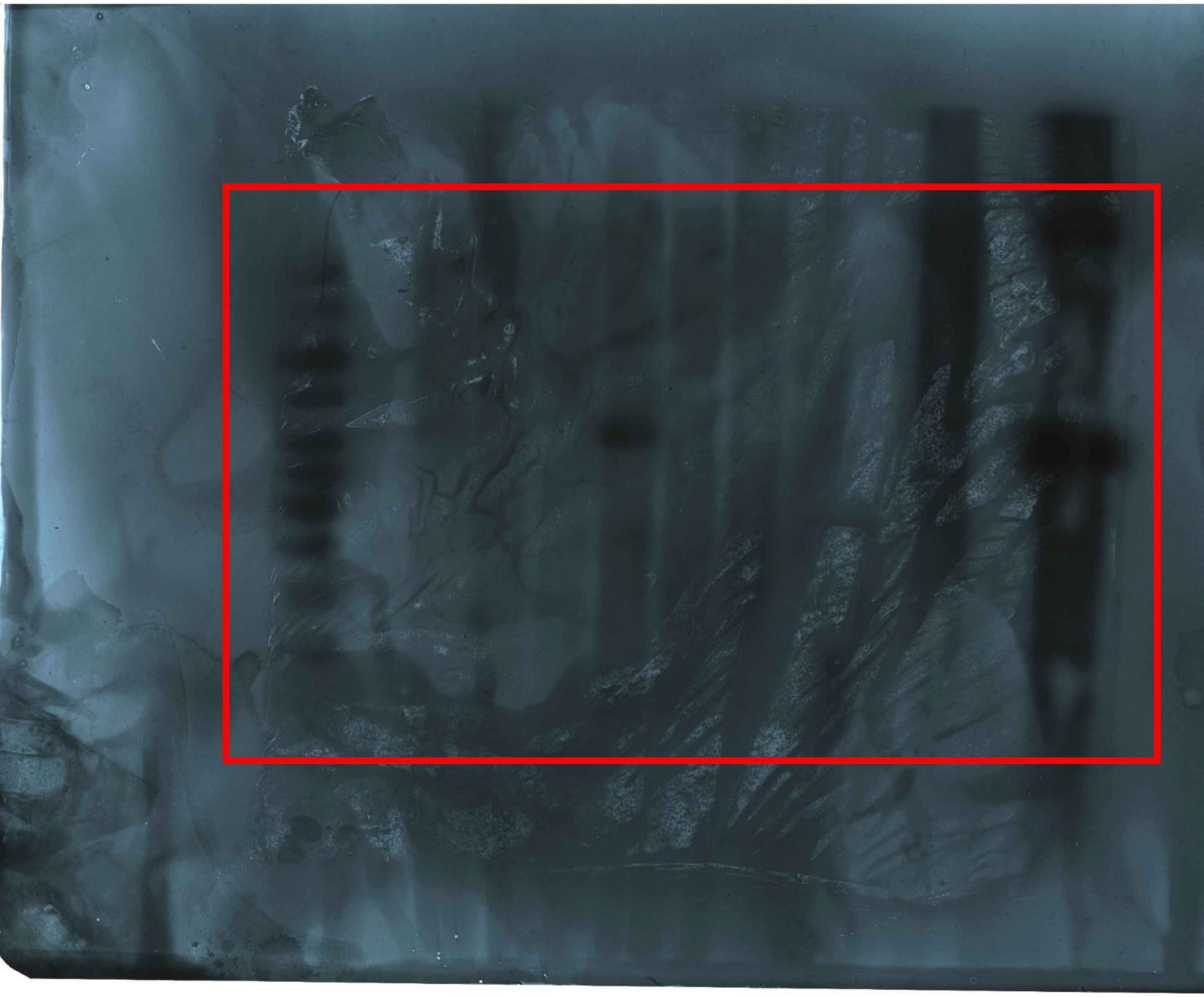

Figure S6A3

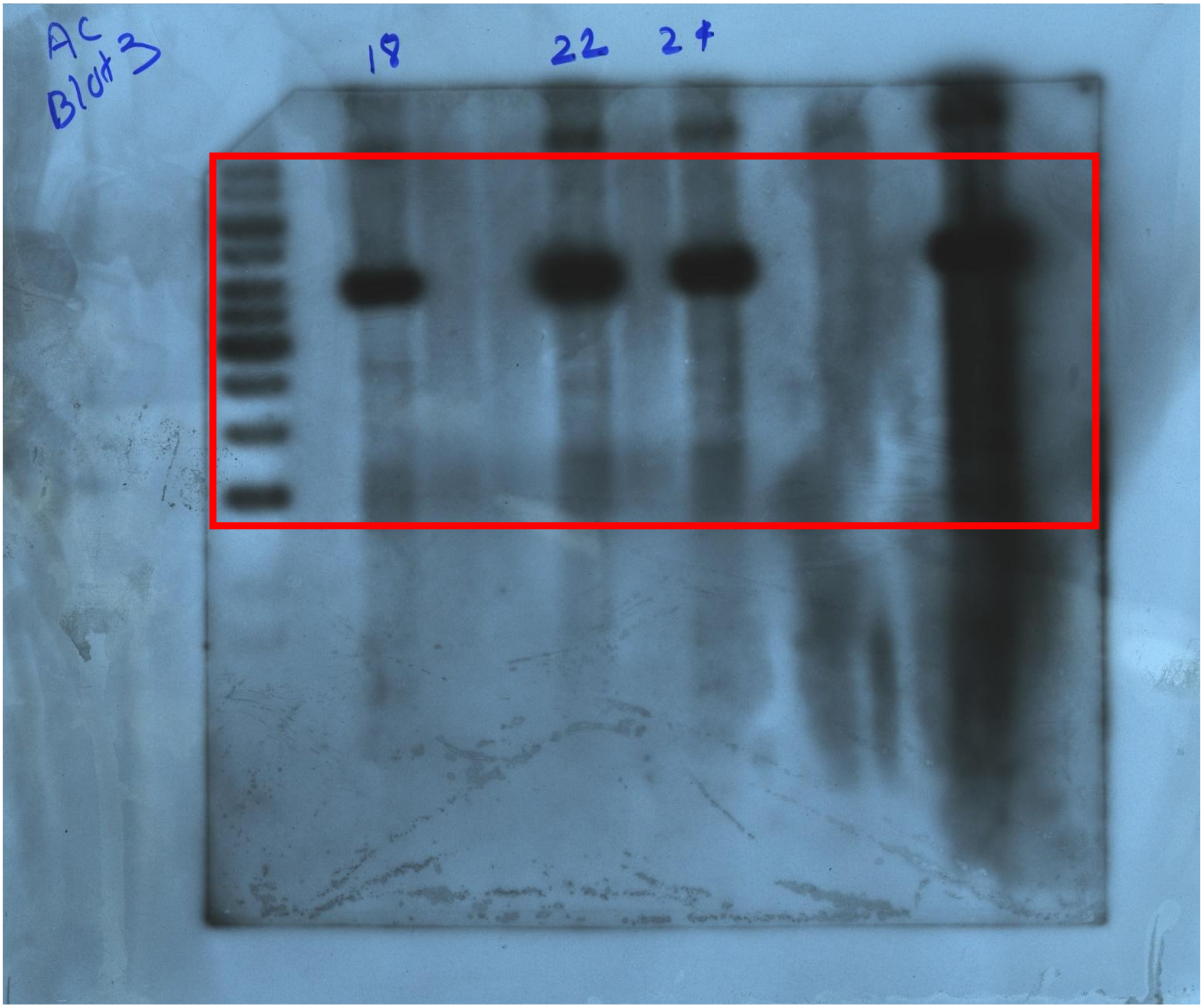

Figure S6B

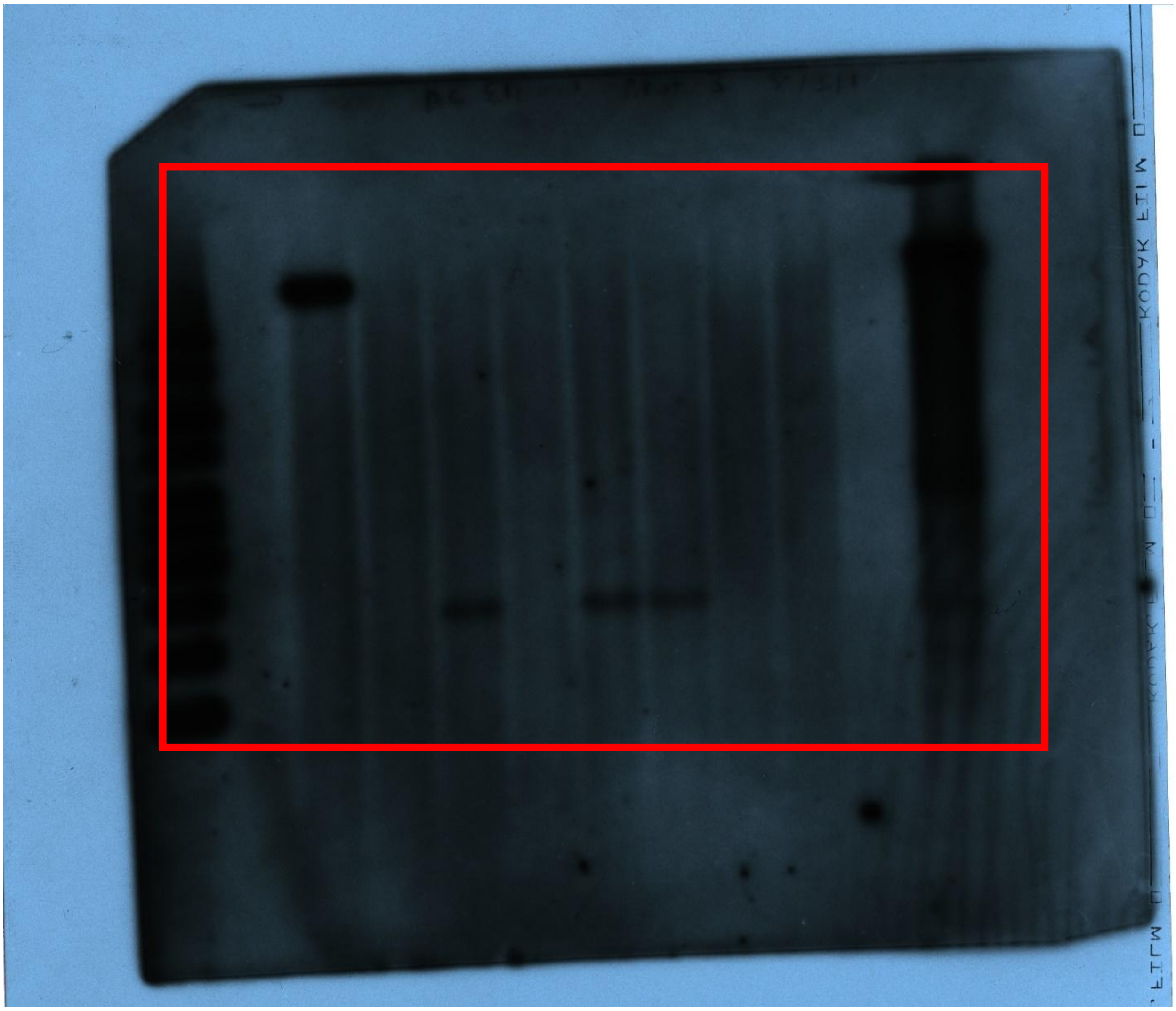

Figure S6C1

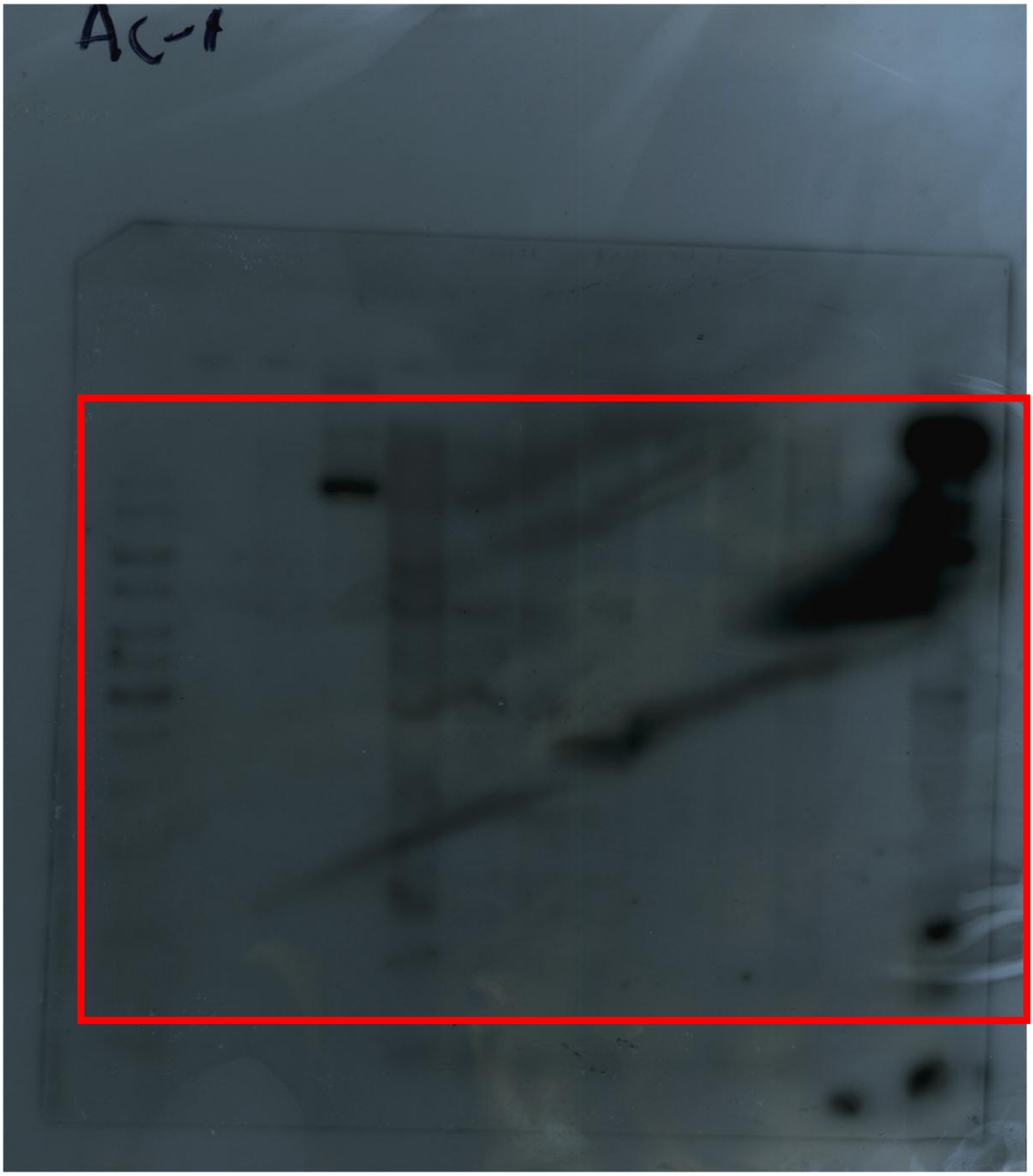

Figure S6C2

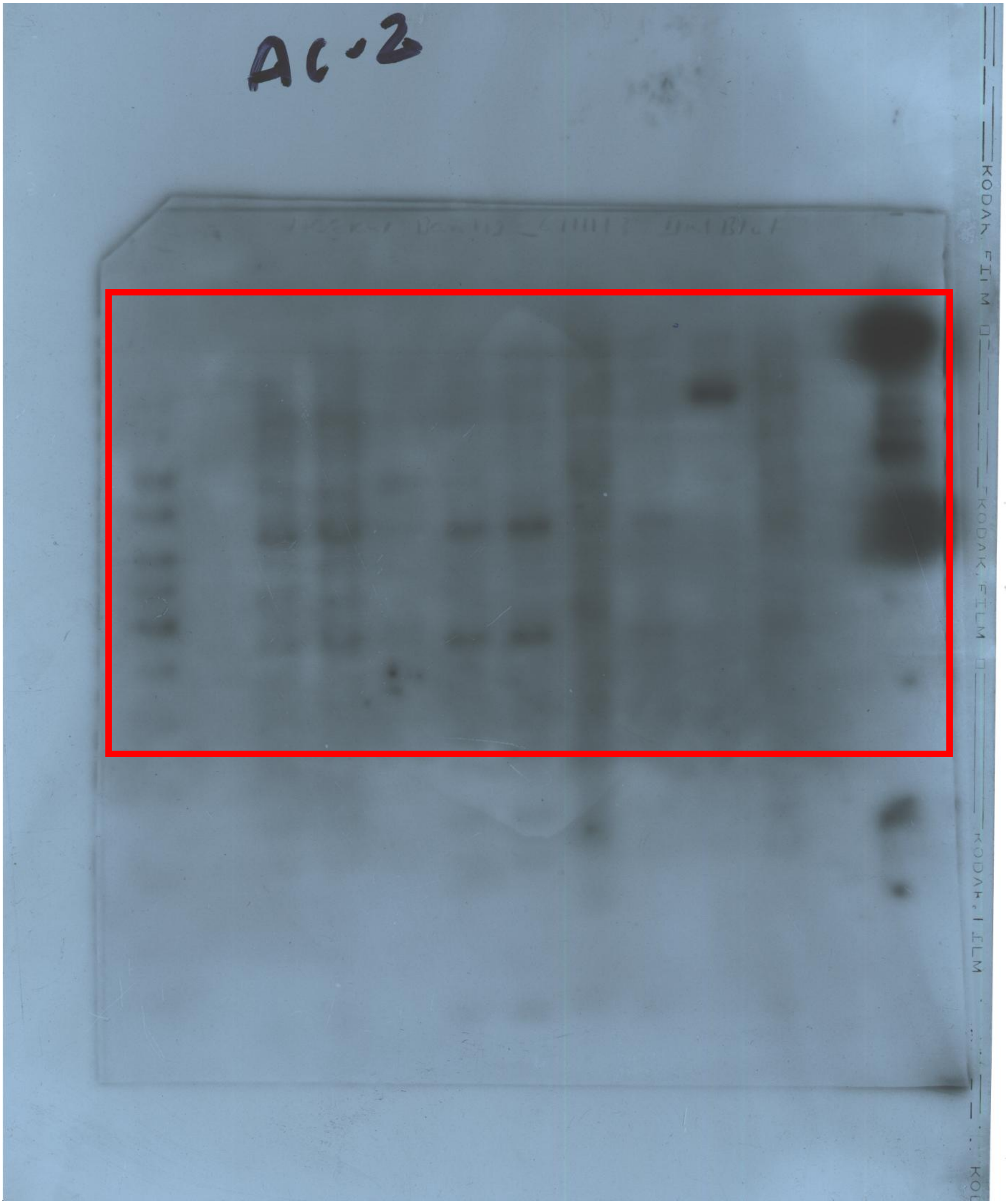

Figure S6C3

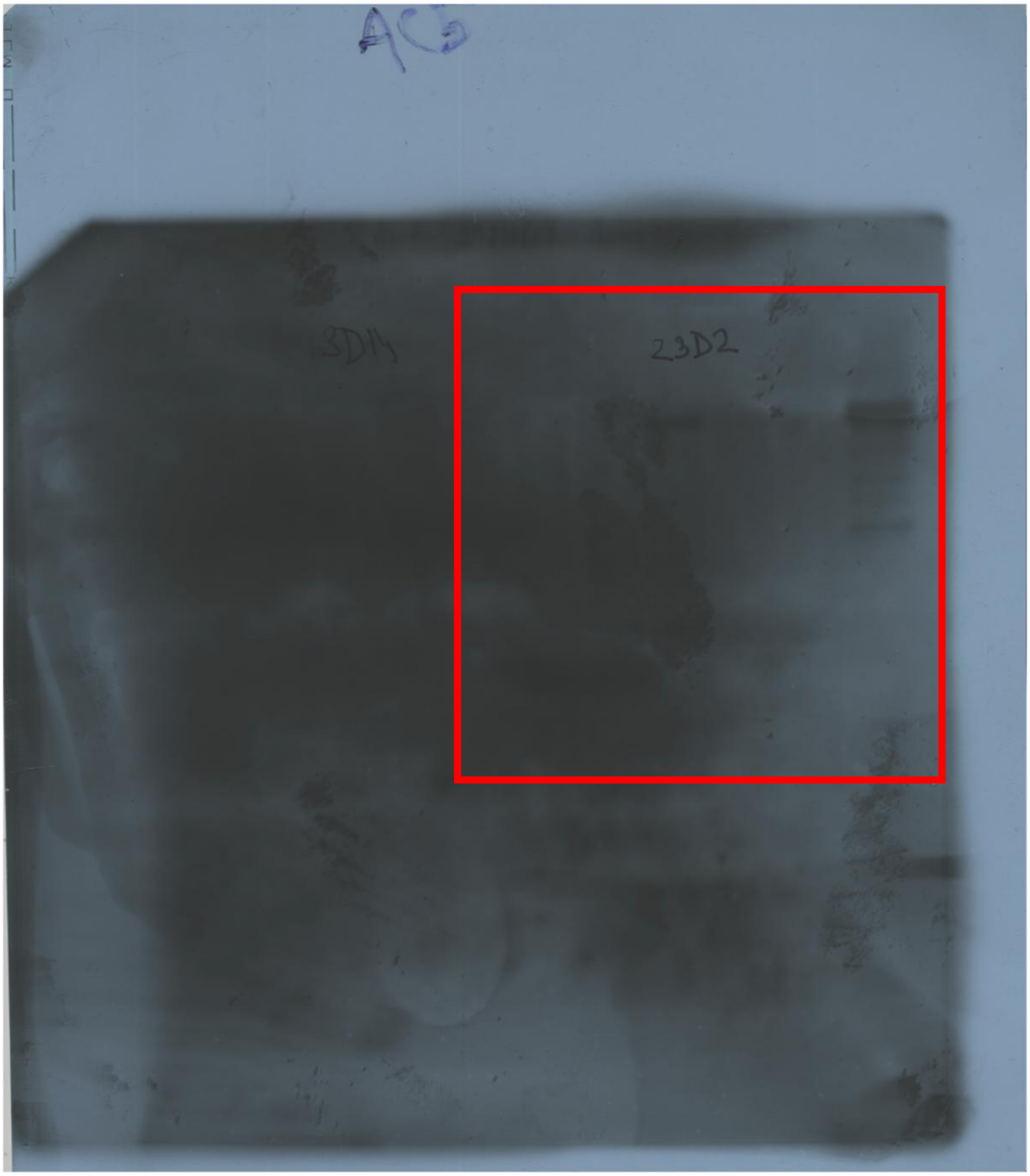

Figure S6C4

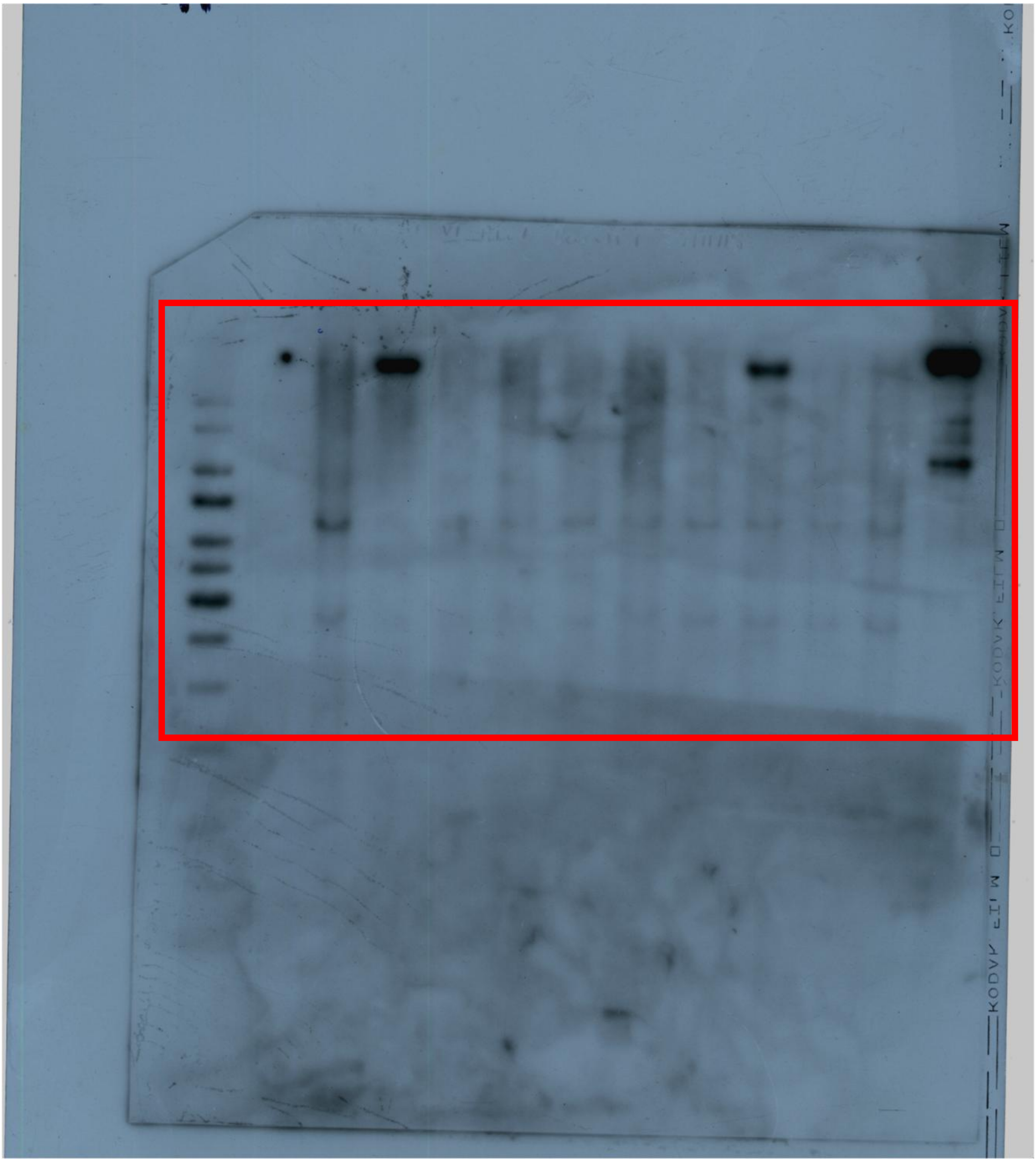

Supplement: S1 File — (PDF) [file pone.0335612.s021.pdf]
