# Supplementary material for: Development of Ac- and Ds-tagged starter lines for large-scale transposon-mutagenesis in tomato
Source: PLoS One. 2025 Nov 19;20(11):e0335612. doi: 10.1371/journal.pone.0335612 (PMC12629433; doi:10.1371/journal.pone.0335612)
Supplement: S7 Fig — (PDF) [file pone.0335612.s007.pdf]

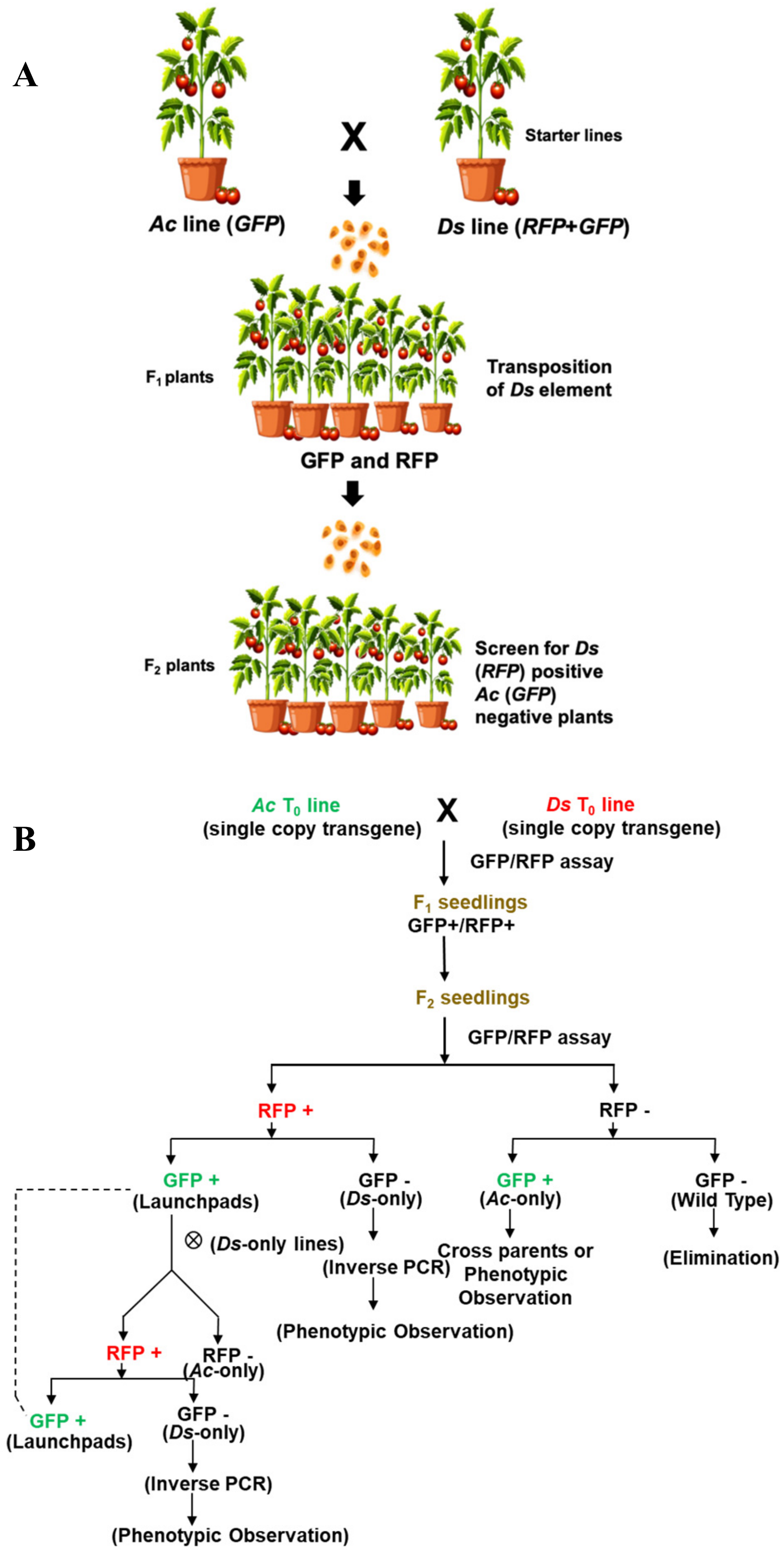

S7 Fig. The screening process for selecting plants with *Ds* transposition. **A.** Graphical illustration of the generation of transposon-tagged mutant population in tomato using the *Ac/Ds* transposon system. **B.** The flowchart outlines the screening process for selecting plants with *Ds* transposition. The presence of both GFP and RFP genes in successive generations is assessed through fluorescence assay and PCR analysis. The F_1 seedlings carrying both *Ac* and *Ds* elements (GFP+/RFP+) are carried forward to the F_2 generation. Within the F_2 population, based on screening, plants are classified into four categories. Plants exhibiting only RFP (GFP-/RFP+) indicate successful transposition of *Ds* into a new site. Plants displaying only GFP (GFP+/RFP-) are identified as *Ac*-only plants. Wild-type plants are characterized by neither GFP nor RFP (GFP-/RFP-). Plants exhibiting both GFP and RFP (GFP+/RFP+) can serve as "launch pads" for identifying new instances of *Ds* transposition, as described above.
